# A Click Chemistry Approach towards Flavin-Cyclodextrin Conjugates—Bioinspired Sulfoxidation Catalysts

**DOI:** 10.3390/molecules201119667

**Published:** 2015-11-04

**Authors:** Petra Tomanová, Jiří Šturala, Miloš Buděšínský, Radek Cibulka

**Affiliations:** 1 Department of Organic Chemistry, University of Chemistry and Technology, Prague, Technická 5, 166 28 Prague 6, Czech Republic; peta.tomanova@gmail.com (P.T.); sturalaj@vscht.cz (J.Š.); 2 Institute of Organic Chemistry and Biochemistry AS CR, v.v.i., Flemingovo nám. 2, 166 10 Prague 6, Czech Republic; budes@uochb.cas.cz

**Keywords:** click chemistry, cyclodextrin, flavin, monooxygenase, oxidation, sulfoxides, green chemistry

## Abstract

A click chemistry approach based on the reaction between alkynylflavins and mono(6-azido-6-deoxy)-β-cyclodextrin has proven to be a useful tool for the synthesis of flavin-cyclodextrin conjugates studied as monooxygenase mimics in enantioselective sulfoxidations.

## 1. Introduction

**Scheme 1:**
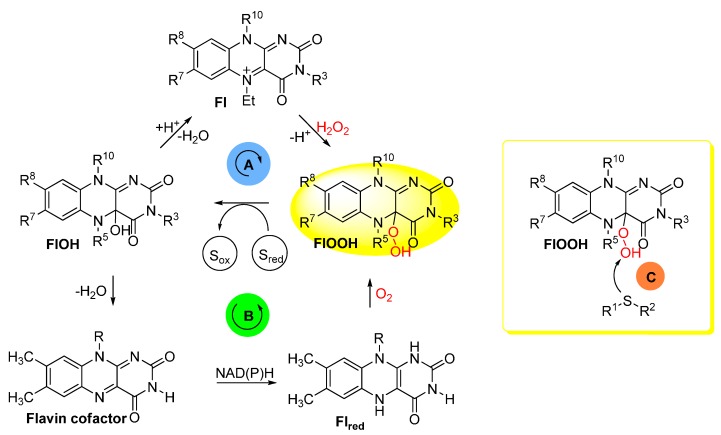
Formation of flavin-4a-hydroperoxide **FlOOH** in H_2_O_2_-sulfoxidations catalyzed by flavinium salts **Fl** (R^5^ = Et) (**A**) and in sulfoxidations taking place in class B monooxygenases (R^5^ = H) (**B**). Oxidation of sulfides by **FlOOH** takes place by electrophilic mechanism (**C**), *i.e.*, by an attack of sulfur to electrophilic hydroperoxy group oxygen.

Within the last three decades, flavinium salts have been shown as useful tools for the organocatalytic activation of hydrogen peroxide and oxygen allowing various oxidations to proceed under mild conditions [1,2,3]. In particular, extensive research on H_2_O_2_-sulfoxidations catalyzed by flavinium salts has resulted in several useful synthetic procedures for mild, chemoselective and stereoselective transformations of sulfides to sulfoxides [4,5,6,7,8,9]. In these artificial systems, flavinium salts **Fl** mimic the function of flavin co-factors in monooxygenases [10] via the *in situ* formation of flavin-4a-hydroperoxide **FlOOH**, which subsequently oxidizes the substrate (Scheme 1).

Flavoenzymes usually give the corresponding oxygenated products chemoselectively and stereoselectively, which is provided by the stereoselective transfer of activated oxygen from flavin-4a-hydroperoxide to the substrate accommodated in an active site of the enzyme via non-covalent bonding interactions [11,12,13,14]. The efficiency of an enzyme is usually sensitive to the modification in its active site as well as to a change in the substrate; both of which can lead to a significant loss of stereoselectivity [12,14,15,16]. Cyclodextrins have been extensively studied as biomimetic catalysts by utilizing their hydrophobic cavity for non-covalent binding of lipophilic substrates in neat aqueous media [17,18,19]. Moreover, analogously to an enzyme active site, the cyclodextrin cavity offers a chiral environment for asymmetric transformations [20,21,22,23,24,25].

**Figure 1:**
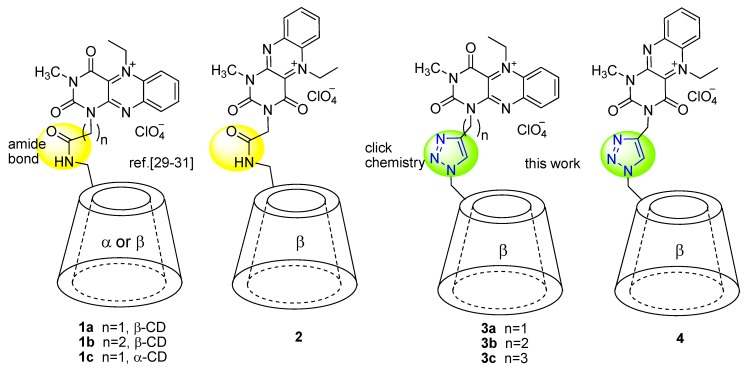
Design of flavin-cyclodextrin conjugates.

Souza and coworkers investigated artificial enzymes based on flavin and cyclodextrin moieties linked together with ether or ester functionality. The conjugates were tested in oxidation of mercaptans, NADH^+^ models and in photooxidation of benzyl alcohols [26,27,28]. Recently, we have designed sulfoxidation catalysts **1** and **2** (Figure 1) containing a redox-active flavinium “co-factor” and chiral substrate-binding site made using α- or β-cyclodextrin; both parts were attached by amide bonds [29,30,31]. The efficiency of these biomimetic catalysts was shown to depend strongly on the structure of the “co-factor”, the relative position of both parts of the catalyst as well as the size of the cyclodextrin cavity. The best sulfoxidation catalysts were found to be alloxazinium-β-cyclodextrin conjugates **1a** and **1b**, which achieved an ee of up to 91% for *tert*-butyl methyl sulfide, 80% ee for aromatic sulfides and up to 64% ee for aliphatic sulfides with only 1 mol % catalyst in neat aqueous media. It was noteworthy that a small change in the catalyst structure such as turning the alloxazine relative to the cyclodextrin cavity (see catalyst **2**) caused the stereoselectivity of the reaction to be completely lost [31].

Herein, we report a new synthetic approach towards flavin-cyclodextrin conjugates based on click chemistry, specifically the copper-catalyzed azide-alkyne [3+2] cycloaddition (CuAAC) [32,33,34,35]. We used the same co-factor (alloxazine) and cavity (β-cyclodextrin) as in **1**, to prove if the CuAAC methodology is compatible with the alloxazine unit since although click chemistry is well established among cyclodextrins [34,36], its use among flavins is still limited to only one isoalloxazine example [37,38].

## 2. Results and Discussion

### 2.1. Synthesis of Conjugates ***3*** and ***4***

**Scheme 2:**
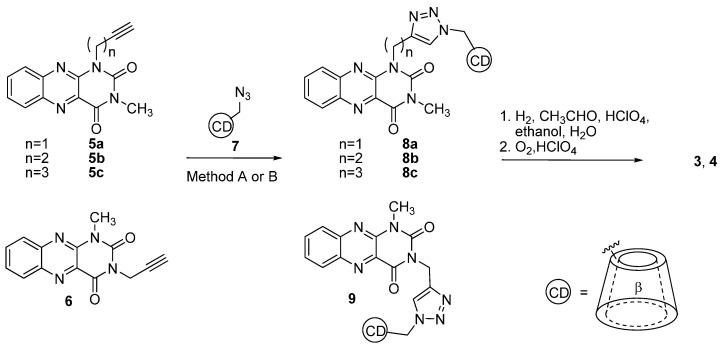
Synthesis of catalysts **3** and **4** using click chemistry approach. Method A: CuI, *N*,*N*-diisopropylethylamine, DMF; method B: TBTA, sodium ascorbate, CuSO_4_, DMF.

The strategy used towards the synthesis of new catalysts **3** and **4** corresponded to the synthetic pathway used for derivatives **1** and **2** (see Scheme 2). The synthetic route started with the preparation of flavin and cyclodextrin subunits functionalized with suitable groups for interconnecting both parts followed by the transformation of the neutral alloxazine into the corresponding alloxazinium moiety. Alkylation/quaternization of the N5 nitrogen is essential for the flavins to be active in the artificial oxygenations. Amide coupling of mono(6-amino-6-deoxy)-β-cyclodextrin with the corresponding flavin carboxylic acid used in the synthesis of **1** and **2** was replaced by a CuAAC between mono(6-azido-6-deoxy)-β-cyclodextrin **7** and an alloxazine possessing a terminal triple bond in its side chain. 1-(Alkynyl)-3-methylalloxazine **5** and 1-methyl-3-propargylalloxazine **6** were prepared by alkylation of 3-methylalloxazine or 1-methylalloxazine, respectively, with a triple-bond containing alkylating agent. Mono(6-azido-6-deoxy)-β-cyclodextrin **7** was readily available using standard procedures starting from commercial β-cyclodextrin [39].

The preliminary tests of the CuAAC reaction between 3-methyl-1-propargylalloxazine **5a** and the model azides (benzylazide or *p-*tolylazide) gave the corresponding triazoles in excellent yield without decomposition of the alloxazine skeleton using conventional reaction conditions [32,33,34,35], *i.e.*, copper iodide and Hünig’s base in *N*,*N*-dimethylformamide (DMF) (see Supplementary Materials). Therefore, this method (A) was applied for the cycloaddition of alkynylalloxazines **5** and **6** with azide **7**. In all cases, the corresponding conjugates were formed in moderate yield with the exception of derivative **8b**, which was not formed using method A despite great effort (Table 1). Lower yields of conjugates could be a result of redox activity of flavin moiety supporting undesired oxidation of Cu(I) during cycloaddition with less reactive azidocyclodextrine **7**. Therefore we modified reaction conditions using copper sulfate, sodium ascorbate (to produce Cu(I) *in-situ*) and the tris[(1-benzyl-1*H*-1,2,3-triazol-4-yl)methyl]amine (TBTA) ligand which is known to stabilize Cu(I) towards disproportionation and oxidation (method B) [40]. Under these conditions, **8b** was formed in good yield. Method B even gave the conjugates **8** and **9** in substantially higher yields, regardless of the position of the alkynyl group on the alloxazine subunit and the length of the spacer (Table 1). Importantly, the triazole spacer tolerated the subsequent reaction steps, which led to the transformation of the neutral alloxazine to alloxazinium moiety, *i.e.*, the introduction of an ethyl group via reductive amination and oxidation, which proceeded in a quantitative yield in all cases.

**Table 1:** Click reaction between flavins **5** or **6** and azidocyclodextrin **7** ^a^.

Entry	Alloxazine	Conjugate	Method	Yield
1	**5a**	**8a**	A	68
2	**5a**	**8a**	B	82
3	**5b**	**8b**	A	- ^b^
4	**5b**	**8b**	B	58
5	**5c**	**8c**	A	23
6	**5c**	**8c**	B	91
7	**6**	**9**	A	34
8	**6**	**9**	B	64

^a^ For procedures, see Experimental Section; ^b^ Product formation was not observed.

### 2.2. Catalytic Activity

To demonstrate the activity of the modified biomimetic systems in a catalytic oxidation, the prepared conjugates were subjected to preliminary screening in the enantioselective sulfoxidation of alkyl methyl sulfides and aryl methyl sulfides using hydrogen peroxide as a model reaction. The oxidation was performed in a buffered aqueous medium under the reaction conditions recently used in reactions catalyzed by analogous conjugates bearing an amide linker. Among the conjugates bearing a triazole spacer, the efficiency in the enantioselective sulfoxidations decreased with an increase in the spacer length and by “turning” the alloxazine moiety going from **3** to **4** (see Supplementary Materials). In Table 2, the reaction conversions and enantioselectivities of the sulfoxidations catalyzed by **3a**, the most efficient conjugate with triazole linker, are compared with the catalytic activity of **1b**, one of the most efficient conjugate from “amide” series with the same length of spacer. The reaction conversions of the non-catalyzed oxidation reactions (blanks) are given for information. The observed enantioselectivities obtained using **3a** were lower than those found when using **1b** but still comparable or even better than those obtained using other flavin-cyclodextrin conjugates or other chiral flavinium catalysts [7,9,29,30,31]. Interestingly, the enantioselectivities obtained using **3a** were relatively high with difficult substrates such as benzyl, hexyl and butyl methyl sulfides, which are usually oxidized with low enantioselectivities. The lower enantioselectivities compared to **1b** are probably a result of the lower rate of reaction found when using **3a**, which is demonstrated by the lower reaction conversions obtained after 1 h; while complete conversion was observed after 60 min when using catalyst **1** [29,30,31], the reaction was not finished when using catalyst **3a**. This fact causes that the non-stereoselective oxidation of the substrate with hydrogen peroxide (blank) competes significantly with the reaction catalyzed by **3a**. This was in accordance with the observation that a higher catalyst loading increased the enantioselectivity of the reactions up to 50% ee in the case of substrates, which are susceptible to non-catalyzed oxidation (e.g., *t*-BuSCH_3_ or BuSCH_3_).

**Table 2:** H_2_O_2_-sulfoxidations catalyzed by conjugate **3a** and comparison with data for **1b** and non-catalyzed reaction ^a^.

Sulfide	Conversion ^b^ (%)/ee ^c^ (%)		Conversion of Blank ^b,d^ (%)
	3a	1b ^e^	
*n*-C_4_H_9_SCH_3_	quant. ^f^/33	-	97
	**quant. ^f^/50 ^g^**		
*n*-C_6_H_13_SCH_3_	42/39	92/64	10
*n*-C_8_H_17_SCH_3_	9/11	44/29	2
*n*-C_10_H_21_SCH_3_	34/10	99/0	0
*t*-C_4_H_9_SCH_3_	quant. ^f^/13	98/86	98
	**quant. ^f^/30 ^g^**		
*c*-C_6_H_11_SCH_3_	77/26	92/80	39
BnSCH_3_	**59/35**	92/58	20
*p*-TolylSCH_3_	36/26	96/69	4
PhSCH_3_	38/20	70/36	2

^a^ Conditions: substrate (0.1 mmol), H_2_O_2_ (2.3 equiv.), phosphate buffer pH 7.5, 25 °C, catalyst loading 1 mol % (related to the substrate) if not stated otherwise; vigorous shaking for 1 h; ^b^ conversion determined by ^1^H-NMR; ^c^ ee determined by HPLC on a chiral stationary phase (see Supplementary Material for details); ^d^ oxidations without catalyst; ^e^ data from ref. [30]; ^f^ quantitative conversion; ^g^ 5 mol % of the catalyst.

The results show the replacement of amide by triazole spacer influences the catalytic activity of flavin-cyclodextrin conjugates. Most probably higher hydrogen bonding ability of amide function compared to triazole could positively influence the efficiency of amide type catalysts **1**. Hydrogen bonds could (i) increase the intrinsic reactivity of hydroperoxide function in flavin-4a-hydroperoxide during oxygen transfer to a substrate (see Figure S6 in Supporting Materials for illustration) [41]; and (ii) affect relative arrangement of flavin and cyclodextrin subunits. Nevertheless, as shown recently [31], the catalytic activity of flavin-cyclodextrin conjugates is very sensitive on only minor changes in their structure and thus, further studies are necessary to fully understand the structure activity relationship.

Although flavin-cyclodextrin conjugates do not achieve enantioselectivities exhibited by enzymes (see Table 3), they seem to be promising biomimetic catalysts of sulfoxidations. They provide the oxidations within a relatively short time and in contrast to enzymes, they usually work at higher substrate concentrations. Biocatalytic systems with monooxygenases are more complicated as they require regeneration of NADPH. Cofactor regeneration is usually provided by glucose-6-phosphate dehydrogenase using glucose-6-phosphate as sacrificial reductant. On the other hand biocatalytic systems use oxygen as stoichiometric oxidant, which, advantageously, avoids any background (non-stereoselective) oxidation without participation of the catalyst since oxygen itself is not reactive enough to oxidize most sulfides under mild conditions. Undesired non-catalyzed oxidation unfortunately occurs in oxidations with hydrogen peroxide (see blank experiments in Table 2) thus decreasing overall stereoselectivity. Probably, use of oxygen in place of hydrogen peroxide in oxidations with flavin-cyclodextrin conjugates, analogously to enzymes (combined with a sacrificial reducing agent [2]), would be a way to eliminate some background non-selective oxidations. The other way to improve our catalytic systems could be to optimize the solvent conditions or to increase the reactivity of flavin subunit by introduction of a strong electron-withdrawing group to flavin benzene ring [6].

**Table 3:** Comparison of the catalytic activity of flavin-cyclodextrin conjugates **1a** and **3a** with enzymes in sulfoxidations of selected sulfides.

Catalyst	Concentration of Substrate (mM)	Reaction Time (h)	Conversion (%)/ee (%)			
			*n*-C_4_H_9_SCH_3_	PhSCH_3_	*p*-TolylSCH_3_	*t*-C_4_H_9_SCH_3_
**1a ^a^**	120	1	-	93/64	99/80	99/88
**3a ^b^**	120	1	quant. ^e^/50	-	-	quant. ^e^/30
**HAPMO ^c^**	10–20	24	99/99 ^f^	96/99	77/99	-
**CHMO ^d^**	35	overnight	-	88/99	94/37	98/99

^a^ 1 mol %, data from ref. [30]; ^b^ 5 mol %; ^c^ Recombinant 4-hydroxyacetophenon monooxygenase from *Pseudomonas fluorescens* ACB, 1 U/mL, data from ref. [42]; ^d^ Cyclohexanone monooxygenase from *Acinetobacter calcoaceticus*, 0.5 U/mL, data from ref. [15]; ^e^ quantitative conversion; ^f^ data from ref. [43].

## 3. Experimental Section

### 3.1. General Information

NMR spectra were obtained on Varian Mercury Plus 300 (^1^H at 299.9 MHz, ^13^C at 75.5 MHz) (Varian, Palo Alto, CA, USA), Agilent 400-MR DDR2 (^1^H at 399.9 MHz, ^13^C at 100.6 MHz) (Agilent Technologies, Santa Clara, CA, USA), Bruker AVANCE 500 (^1^H at 500.1 MHz, ^13^C at 125.8 MHz) (Bruker Bioscience, Billerica, MA, USA) and Bruker AVANCE 600 (^1^H at 600.1 MHz, ^13^C at 150.9 MHz) spectrometers (Bruker Bioscience). Mass spectra were acquired using ES ionization on LTQ Orbitrap XL spectrometer (ThermoFisher Scientific Inc., Waltham, MA, USA). FT-IR spectra were measured on Nicolet 6700 equipped with ATR accessory (ThermoFisher Scientific Inc.), UV-VIS spectra on Varian Cary 50 spectrometer (Varian). Enantiomeric excess was determined by HPLC on columns with chiral stationary phase equipped with UV detector (wavelength 254 nm and 215 nm; Ingos, Prague, Czech Republic). TLC and RP-TLC chromatographies were performed with percoated Silica Gel DC Alufolien Kieselgel 60 F_254_ and 60 RP-18 F_254_ plates from Merc (Merck-Schuchardt, Hohenbrunn, Germany). Melting points were determined on Boetius microscopic apparatus (Hebenstreit-Rapido GmbH, Radebeul, Germany). Optical rotation was measured on Perkin-Elmer polarimeter 241 (PerkinElmer, Akron, OH, USA).

Alkylating agents 4-bromobut-1-yne [44] and 5-bromopent-1-yne [45,46,47], and alloxazines 3-methylalloxazine [48] and 1-methylalloxazine [31] were prepared according to previously published procedures.

### 3.2. Synthesis of Conjugates

#### 3.2.1. Alkylation of 3-Methylalloxazine

##### General Procedure

Substituted alloxazine was dissolved in dry *N*,*N*-dimethylformamide (DMF) and 2 equivalents of alkynylbromide and 5 equivalents of potassium carbonate were added. Reaction mixture was stirred under nitrogen atmosphere for 24 h. The reaction was followed by TLC (chloroform/methanol 100:3). Solid salts were filtered off and solvent was evaporated *in vacuo*. Residue was dissolved in dichloromethane and washed with water. Water layer was separated and extracted with dichloromethane. Collected organic layers were washed with water and dried with sodium sulfate. After evaporation of solvents, solid product was dried *in vacuo*.

*3-Methyl-1-propargylalloxazine* (**5a**): Synthesis according to general procedure with 3-methylalloxazine (0.5 g, 2.2 mmol), propargylbromide (0.7 g, 4.4 mmol) and potassium carbonate (1.5 g, 11.0 mmol). **5a** was obtained as yellow solid (0.5 g, 79%). M.p. 241–244 °C. ^1^H-NMR (300 MHz, CDCl_3_) δ 8.37 (dd, *J* = 8.4, 1.0 Hz, 1H), 8.10 (dd, *J* = 8.4 Hz, 1H), 7.93 (ddd, *J* = 8.5, 6.8, 1.5 Hz, 1H), 7.80 (ddd, *J* = 8.4, 6.9, 1.4 Hz, 1H), 5.26 (d, *J* = 2.4 Hz, 2H), 3.63 (s, 3H), 2.23 (t, *J* = 2.5 Hz, 1H), ^13^C-NMR (75 MHz, CDCl_3_) δ 159.28, 149.60, 143.62, 142.87, 140.04, 133.77, 130.52, 129.23, 127.72, 71.18, 31.68, 28.98. HRMS-ESI^+^ *m*/*z*: [M + Na]^+^ calcd for C_14_H_10_N_4_NaO_2_ 289.0696, found 289.0696.

*1-(But-3-ynyl)-3-methylalloxazine* (**5b**): Synthesis according to general procedure with 3-methylalloxazine (1.0 g, 4.4 mmol), 4-brombut-1-yn (1.1 g, 8.8 mmol) and potassium carbonate (3.0 g, 22.0 mmol). **5b** was obtained (1.2 g, 95%) as yellow solid. M.p. 260–261 °C. ^1^H-NMR (300 MHz, CDCl_3_) δ 8.39–8.32 (m, 1H), 8.09–8.02 (m, 1H), 7.92 (ddd, *J* = 8.5, 6.9, 1.5 Hz, 1H), 7.78 (ddd, *J* = 8.4, 6.8, 1.4 Hz, 1H), 4.69 (t, *J* = 7.3 Hz, 2H), 3.61 (s, 3H), 2.78 (td, *J* = 7.3, 2.7 Hz, 2H), 1.97 (t, *J* = 2.7 Hz, 1H), ^13^C-NMR (75 MHz, CDCl_3_) δ 159.96, 150.50, 146.94, 145.56, 143.42, 140.41, 134.19, 131.04, 129.56, 128.14, 81.41, 70.56, 41.09, 29.45, 17.67. HRMS-ESI^+^ *m*/*z*: [M + Na]^+^ calcd for C_15_H_12_N_4_NaO_2_ 303.0858, found 303.0855.

*3-Methyl-1-(pent-4-ynyl)alloxazine* (**5c**): Synthesis according to general procedure with 3-methylalloxazine (39 mg, 0.17 mmol), 5-brompent-1-yn (50 mg, 0.34 mmol) and potassium carbonate (118 mg, 0.85 mmol). **5c** was obtained (45 mg, 90%) as yellow solid. M.p. 262–264 °C. ^1^H-NMR (300 MHz, CDCl_3_) δ 8.35 (d, *J* = 7.9 Hz, 1H), 8.04 (d, *J* = 8.5 Hz, 1H), 7.95–7.87 (m, 1H), 7.81–7.72 (m, 1H), 4.60 (t, 2H), 3.61 (s, 3H), 2.38 (td, *J* = 6.9, 2.5 Hz, 2H), 2.18–2.02 (m, 3H), 1.95 (t, *J* = 2.7 Hz, 1H), ^13^C-NMR (75 MHz, CDCl_3_) δ 150.83, 145.28, 143.70, 140.41, 134.06, 131.02, 129.42, 128.14, 98.52, 83.50, 68.99, 42.26, 34.87, 29.40, 26.46, 16.55. HRMS-ESI^+^ *m*/*z*: [M]^+^ calcd C_16_H_14_N_4_NaO_2_^+^ 317.1014, found 317.1011.

*1-Methyl-3-propargylalloxazine* (**6**): Synthesis according to general procedure with 1-methylalloxazine (0.5 g, 2.2 mmol), propargylbromide (0.7 g, 4.4 mmol) and potassium carbonate (1.5 g, 11.0 mmol). **6** was obtained (0.4 g, 69%) as yellow solid. M.p. 186–187 °C. ^1^H-NMR (300 MHz, CDCl_3_) δ 8.43–8.27 (m, 1H), 8.11–7.99 (m, 1H), 7.92 (ddd, *J* = 8.5, 6.8, 1.5 Hz, 1H), 7.78 (ddd, *J* = 8.4, 6.8, 1.5 Hz, 1H), 4.97 (d, *J* = 2.5 Hz, 2H), 3.85 (s, *J* = 3.7 Hz, 3H), 2.26–2.23 (m, 1H). ^13^C-NMR (101 MHz, CDCl_3_) δ 159.01, 149.96, 145.55, 143.39, 140.07, 134.13, 130.85, 129.69, 129.36, 127.89, 77.58, 71.43, 31.69, 29.70. HRMS-ESI^+^ *m*/*z*: [M + Na]^+^ calcd for C_14_H_10_N_4_NaO_2_ 289.0696, found 289.0696.

#### 3.2.2. Click Reactions

##### General Procedure A

The mixture of alkynyl alloxazine **5** or **6** and 6-azido-6-deoxy-β-cyclodextrin **7** (1.2 equiv.) was stirred with copper (I) iodide (3 mol %) and *N*,*N*-diisopropylethylamine (2 equiv.) in dry *N*,*N-*dimethylformamide (DMF) at room temperature for 24 h. Conjugate was precipitated after addition of acetone and separated by centrifugation. Crude product was purified by flash chromatography on the column with reversed phase (gradient MeOH/H_2_O 1:9→MeOH/H_2_O 2:3). The obtained solid product **8** or **9** was dried *in vacuo*.

##### General Procedure B

The mixture of alkynyl alloxazine **5** or **6** and 6-azido-6-deoxy-β-cyclodextrin **7** (1.2 equiv.) was stirred with TBTA (tris[(1-benzyl-1*H*-1,2,3-triazol-4-yl)methyl]amine) (20 mol %), sodium ascorbate (20 mol %) and copper (II) sulfate (10 mol %) in dry *N*,*N-*dimethylformamide (DMF) at room temperature for 24 h. Conjugate was precipitated after addition of acetone and separated by centrifugation. Crude product was purified by flash chromatography on the column with reversed phase (gradient MeOH/H_2_O 1:9→MeOH/H_2_O 2:3). The obtained solid product **8** or **9** was dried *in vacuo*.

*1-{[1-(6-Deoxy-β-cyclodextrin-6-yl)-1H-1,2,3-triazol-4-yl]methyl}-3-methylalloxazine* (**8a**): Synthesis according to general procedure A for click reactions using 3-methyl-propargylalloxazine (75 mg, 0.28 mmol), 6-azido-6-deoxy-β-cyclodextrin (390 mg, 0.34 mmol), copper (I) iodide (3 mg, 0.01 mmol), *N*,*N*-diisopropylethylamine (98 µL, 0.56 mmol) and 4 mL of dry DMF. Product **8a** (270 mg, 68%) was obtained as yellowish powder.

Synthesis according to general procedure B for click reactions using 3-methyl-propargylalloxazine (28 mg, 0.11 mmol), 6-azido-6-deoxy-β-cyclodextrin (164 mg, 0.14 mmol), TBTA (11 mg, 0.02 mmol), sodium ascorbate (4 mg, 0.02 mmol), copper (II) sulfate (3 mg, 0.01 mmol), 4 mL of dry DMF. Product **8a** (123 mg, 83%) was obtained as yellowish powder. M.p. 238–239 °C. ^1^H-NMR see Tables S2 and S4, ^13^C-NMR see Tables S1 and S3, HRMS-ESI^+^ *m*/*z*: [M + Na]^+^ calcd for C_56_H_79_N_7_NaO_36_ 1448.4458, found 1448.4456. ${\text{[α]}}_{D}^{20}$ = 105.2° (*c* = 0.107 g/100 mL, H_2_O).

*1-{[1-(6-Deoxy-*β*-cyclodextrin-6-yl)-1H-1,2,3-triazol-4-yl]ethyl}-3-methylalloxazine* (**8b**): Synthesis according to general procedure B for click reactions using 1-but-3-ynyl-3-methylalloxazine (30 mg, 0.11 mmol), 6-azido-6-deoxy-β-cyclodextrin **7** (164 mg, 0.14 mmol), TBTA (11 mg, 0.02 mmol), sodium ascorbate (4 mg, 0.02 mmol), copper (II) sulfate (3 mg, 0.01 mmol), 4 mL of dry DMF. Product **8b** (90 mg, 58%) was obtained as yellowish powder. M.p. 241–242 °C. ^1^H-NMR see Tables S2 and S4, ^13^C-NMR see Tables S1 and S3, HRMS-ESI^+^ *m*/*z*: [M + Na]^+^ calcd for C_57_H_81_N_7_NaO_36_ 1462.4615, found 1462.4615. ${\text{[α]}}_{D}^{20}$ = 104.6° (*c* = 0.108 g/100 mL, H_2_O).

*1-{[1-(6-Deoxy-*β*-cyclodextrin-6-yl)-1H-1,2,3-triazol-4-yl]propyl}-3-methylalloxazine* (**8c**): Synthesis according to general procedure A for click reactions using 3-methyl-1-pent-4-ynylalloxazine (35 mg, 0.14 mmol), 6-azido-6-deoxy-β-cyclodextrin **7** (166 mg, 0.14 mmol), copper (I) iodide (1 mg, 0.01 mmol), *N*,*N*-diisopropylethylamine (41 µL, 0.24 mmol) and 2 mL of dry DMF. Product **8c** (40 mg, 23%) was obtained as yellowish powder.

Synthesis according to general procedure B for click reactions using 3-methyl-1-pent-4-ynylalloxazine (35 mg, 0.12 mmol), 6-azido-6-deoxy-β-cyclodextrin **7** (182 mg, 0.15 mmol), TBTA (12 mg, 0.02 mmol), sodium ascorbate (4 mg, 0.02 mmol), copper (II) sulfate (3 mg, 0.01 mmol), 4 mL of dry DMF. Product **8c** (157 mg, 91%) was obtained as yellowish powder. M.p. 272–274 °C. ^1^H-NMR see Tables S2 and S4, ^13^C-NMR see Tables S1 and S3, HRMS-ESI^+^ *m*/*z*: [M + H]^+^ calcd for C_58_H_84_N_7_O_36_ 1454.4952, found 1454.4958. ${\text{[α]}}_{D}^{20}$ = 112.3° (*c* = 0.110 g/100 mL, H_2_O).

*3-{[1-(6-Deoxy-*β*-cyclodextrin-6-yl)-1H-1,2,3-triazol-4-yl]methyl}-1-methylalloxazine* (**9**): Synthesis according to general procedure A for click reactions using 1-methyl-3-propargylalloxazine (25 mg, 0.07 mmol), 6-azido-6-deoxy-β-cyclodextrin **7** (130 mg, 0.11 mmol), copper (I) iodide (1 mg, 0.01 mmol), *N*,*N*-diisopropylethylamine (33 µL, 0.19 mmol) and 1 mL of dry DMF. Yellowish product **9** (46 mg, 34%) was obtained.

Synthesis according to general procedure B for click reactions using 1-methyl-3-propargylalloxazine (56 mg, 0.21 mmol), 6-azido-6-deoxy-β-cyclodextrin **7** (328 mg, 0.29 mmol), TBTA (22 mg, 0.04 mmol), sodium ascorbate (8 mg, 0.04 mmol), copper (II) sulfate (6 mg, 0.02 mmol), 4 mL of dry DMF. Product **9** (192 mg, 64%) was obtained as yellowish powder. M.p. 288–290 °C. ^1^H-NMR see Tables S2 and S4, ^13^C-NMR see Tables S1 and S3, HRMS-ESI^+^ *m*/*z*: [M + Na]^+^ calcd for C_56_H_79_N_7_NaO_36_ 1448.4458, found 1448.4434. ${\text{[α]}}_{D}^{20}$ = 90.6° (*c* = 0.102 g/100 mL, H_2_O).

#### 3.2.3. Quaternization of Conjugates

##### General Procedure

Conjugate **8** or **9** was mixed with acetaldehyde (300 equiv.), palladium on carbon (10% *w*/*w*), ethanol, water and 0.1 M perchloric acid. Reaction mixture was stirred in autoclave for 21 h under hydrogen atmosphere (0.6 MPa). Palladium was filtered off and organic solvents were evaporated *in vacuo*. Residue was diluted with water to adjust the volume to exactly 2 mL.

Despite our great efforts, we were unable to record clear NMR spectra of the quaternized conjugates due to peak broadening as a result of the presence of radical species [29,30,31]. Nevertheless, in mass spectra only signals of alloxazinium ions were found. UV spectra of all the prepared catalysts showed maxima at approximately 445 and 382 nm, which are characteristic for alloxazinium salts. For details, see Supplementary Materials.

1*-{[1-(6-Deoxy-*β*-cyclodextrin-6-yl)-1H-1,2,3-triazol-4-yl]methyl}-3-methylalloxazinium perchlorate* (**3a**): Synthesis according to general procedure for quarternisation of conjugates using conjugate **8a** (15 mg, 10 µmol), acetaldehyde (170 µL, 3 mmol), palladium on carbon (2 mg, 10% *w*/*w*), ethanol (800 µL), perchloric acid (800 µL, 0.1 M), water (600 µL). HRMS-ESI^+^ *m*/*z*: [M + H]^+^ calcd for C_58_H_84_N_7_O_36_ 1454.4952, found 1454.4955, UV-VIS (pH = 3.6, *c* = 5.0 × 10^−5^ mol/L): 382 nm, 441 nm.

*1-{[1-(6-Deoxy-*β*-cyclodextrin-6-yl)-1H-1,2,3-triazol-4-yl]ethyl}-3-methylalloxazinium perchlorate* (**3b**): Synthesis according to general procedure for quarternisation of conjugates using conjugate **8b** (11 mg, 7 µmol), acetaldehyde (120 µL, 2 mmol), palladium on carbon (2 mg, 10% *w*/*w*), ethanol (570 µL), perchloric acid (570 µL, 0.1 M) and water (430 µL). HRMS-ESI^+^ *m*/*z*: [M + H]^+^ calcd for C_59_H_86_N_7_O_36_ 1468.5109, found 1468.5122. UV-VIS (pH = 3.6, *c* = 3.6 × 10^−5^ mol/L): 382 nm, 448 nm.

*1-{[1-(6-Deoxy-*β*-cyclodextrin-6-yl)-1H-1,2,3-triazol-4-yl)propyl)-3-methylalloxazinium perchlorate* (**3c**): Synthesis according to general procedure for quarternisation of conjugates using conjugate **8c** (12 mg, 8 µmol), acetaldehyde (130 µL, 2 mmol), palladium on carbon (2 mg, 10% *w*/*w*), ethanol (620 µL), perchloric acid (620 µL, 0.1 M) and water (460 µL). HRMS-ESI^+^ *m*/*z*: [M + H]^+^ calcd for C_60_H_88_N_7_O_36_ 1482.5265, found 1482.5272. UV-VIS (pH = 4.1, *c* = 6.2 × 10^−5^ mol/L): 382 nm, 448 nm.

*3-{[1-(6-Deoxy-*β*-cyclodextrin-6-yl)-1H-1,2,3-triazol-4-yl]methyl}-1-methylalloxazinium perchlorate* (**4**): Synthesis according to general procedure for quarternisation of conjugates using conjugate **9** (45 mg, 30 µmol), acetaldehyde (510 µL, 9 mmol), palladium on carbon (7 mg, 10% *w*/*w*), ethanol (2.4 mL), perchloric acid (2.4 mL, 0.1 M) and water (1.8 mL). HRMS-ESI^+^ *m*/*z*: [M + H]^+^ calcd for C_58_H_84_N_7_O_36_ 1454.4952, found 1454.4954, UV-VIS (pH = 4.3, *c* = 1.6 × 10^−5^ mol/L): 385 nm, 448 nm.

### 3.3. Catalytic Oxidations

Catalytic oxidations of alkyl or aryl methyl sulfides were performed in 1 mL thick-walled screw-capped vial (Supelco). The reaction mixtures were prepared by addition of sodium-phosphate buffer (pH = 7.5, 0.05 M, 300 μL), liquid substrate (3 × 10^−5^ mol), appropriate volume of catalyst solution (1 mol %–5 mol %) and finally aqueous hydrogen peroxide (2.3 mol equiv.). Stirring of the reaction mixture was provided by wrist action shaker (900 rpm) for 1 h at 25 °C. Then the reaction was quenched by addition of a solution of sodium dithionite in water (1.4 M, 170 μL). The mixture was extracted with CDCl_3_ (3 × 0.5 mL). Collected organic layers were dried with molecular sieves (3Å) and used for ^1^H-NMR determination of the conversion. Enantiomeric excess was determined by HPLC analysis on column with chiral stationary phase of the sample obtained by evaporation of CDCl_3_ and dissolving a residue in heptane (0.5 mL).

## 4. Conclusions

We have demonstrated the CuAAC concept is useful for the preparation of flavin-cyclodextrin conjugates. The click chemistry represents alternative approach to the original procedure based on amide coupling and seems to be slightly more advantageous especially by reducing the number of reaction steps and improving the overall yield. Recently, Rotello found the isoalloxazine ring tolerates “click chemistry” when preparing flavin-polymer conjugates [37,38]; we have shown the CuAAC approach was tolerated by alloxazines. Therefore, it seems CuAAC can be used as a general tool for the construction of flavin (isoalloxazin or alloxazine) based supramolecular systems, which can be useful not only in organocatalysis, but also in photocatalysis [3,26,27,28] and molecular recognition [49]. The new conjugates show lower efficiency in the enantioselective sulfoxidation reaction when compared with catalyst **1**, which possesses an amide linker. However, **3a** is comparable with the other chiral flavin catalysts [1,2,3] and even overcomes efficiency of flavin conjugates with amide linker in butyl methyl sulfide oxidation achieving ee up to 50%. One should bear in mind that cyclodextrin-based catalysts usually achieve rather moderate enantioselectivities and the results found with conjugate **1** are rather exceptional. The extreme sensitivity to small structural changes observed among the flavin-cyclodextrin conjugates is a typical phenomenon that also occurs in the natural enzymes and its elucidation is currently being investigated in our laboratory.

## Supplementary Materials

Supplementary materials can be accessed at: http://www.mdpi.com/1420-3049/20/11/19667/s1.
