# Supplementary material for: A Click Chemistry Approach towards Flavin-Cyclodextrin Conjugates—Bioinspired Sulfoxidation Catalysts
Source: Molecules. 2015 Nov 4;20(11):19837–48. doi: 10.3390/molecules201119667 (PMC6331787; doi:10.3390/molecules201119667)
Supplement: Supplementary file 1 [file molecules-20-19667-s001.pdf]

molecules

Supplementary Informations

1. NMR and IR spectra of alloxazine precursors 5 and 6 S2
2. Model experiments for click-chemistry with flavins S8
3. Characterization of flavin-cyclodextrin conjugates 8 and 9 S10
4. Syntesis and characterization of flavinium catalysts 3 and 4 S22
5. Details on stereoselective sulfoxidations S26
6. Effect of amide bond on the reactivity of flavin-4a-hydroperoxide S31

1. NMR and IR Spectra of Alloxazine Precursors 5 and 6

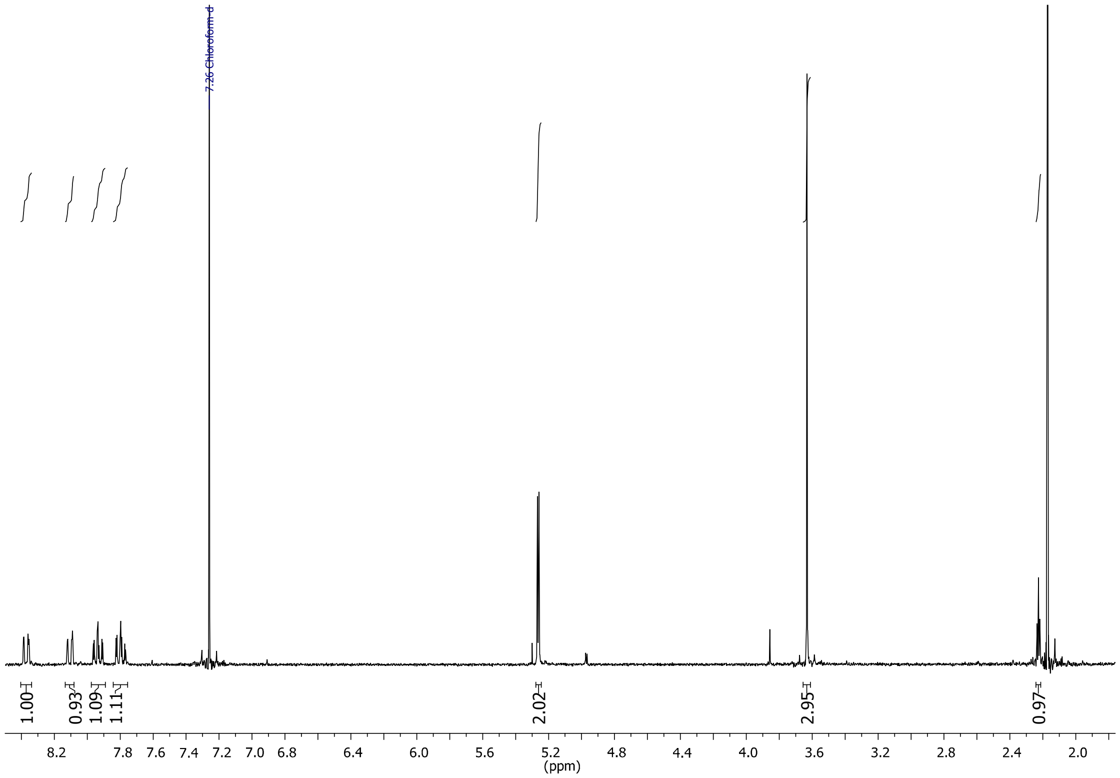

Figure S1. ^{1}H-NMR of compound **5a**.

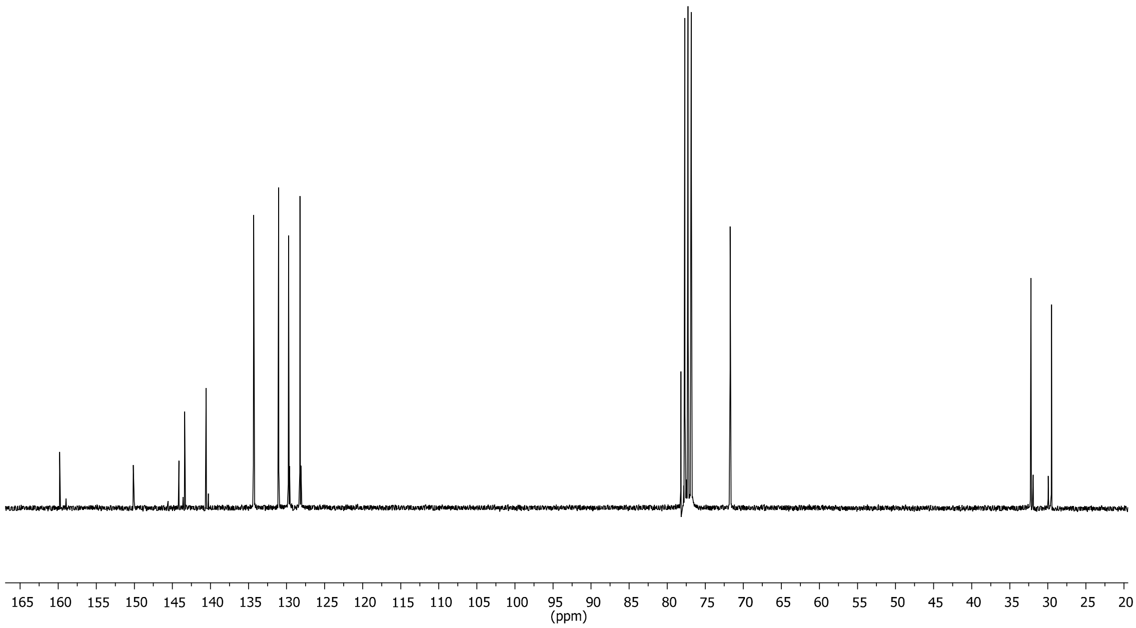

Figure S2. ^{13}C-NMR of compound **5a**.

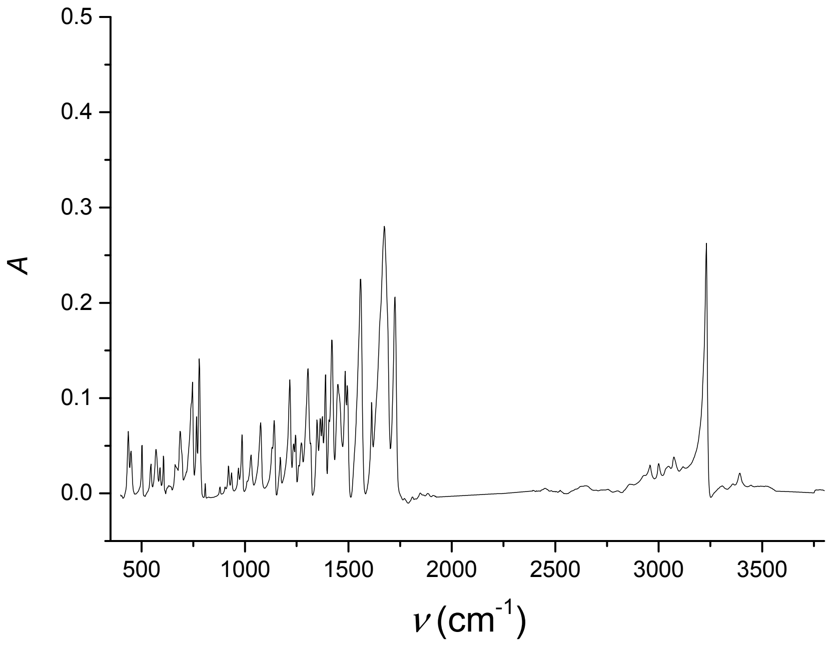

Figure S3. IR of compound **5a**.

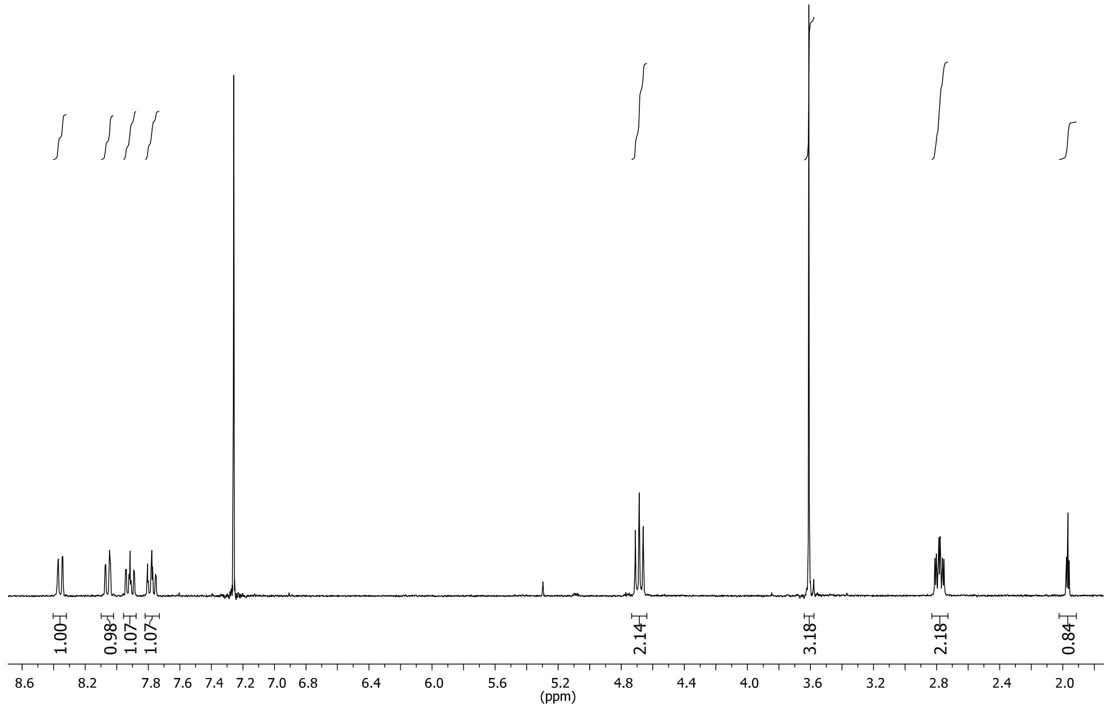

Figure S4. ^{1}H-NMR of compound **5b**.

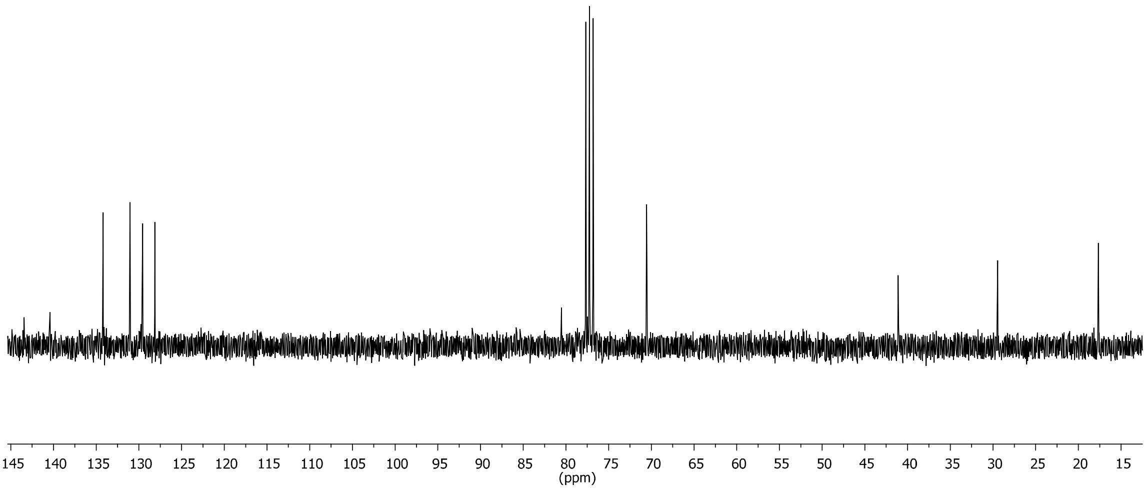

Figure S5. ^{13}C-NMR of compound **5b**.

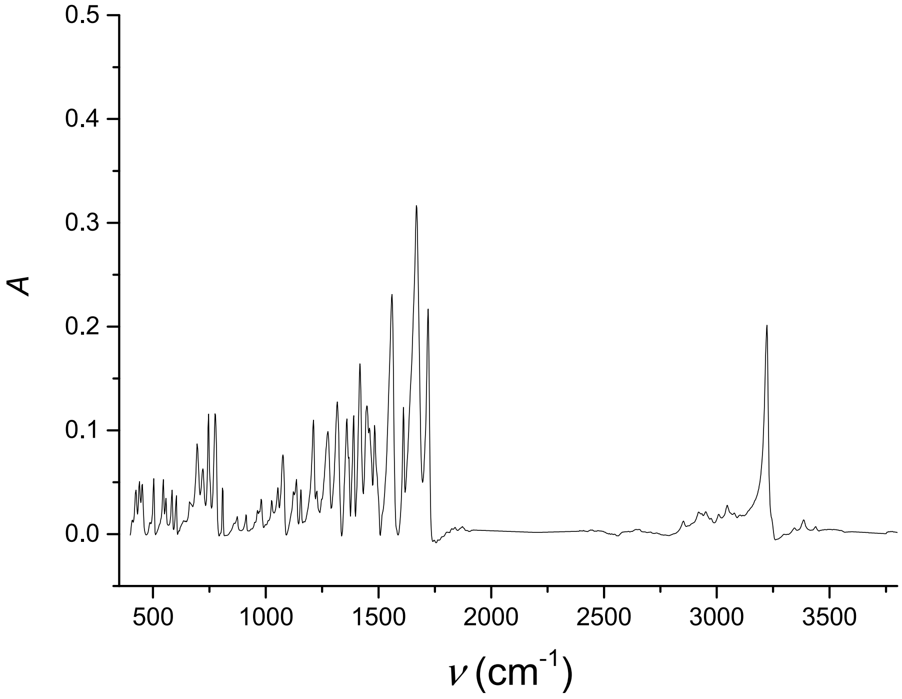

Figure S6. IR of compound **5b**.

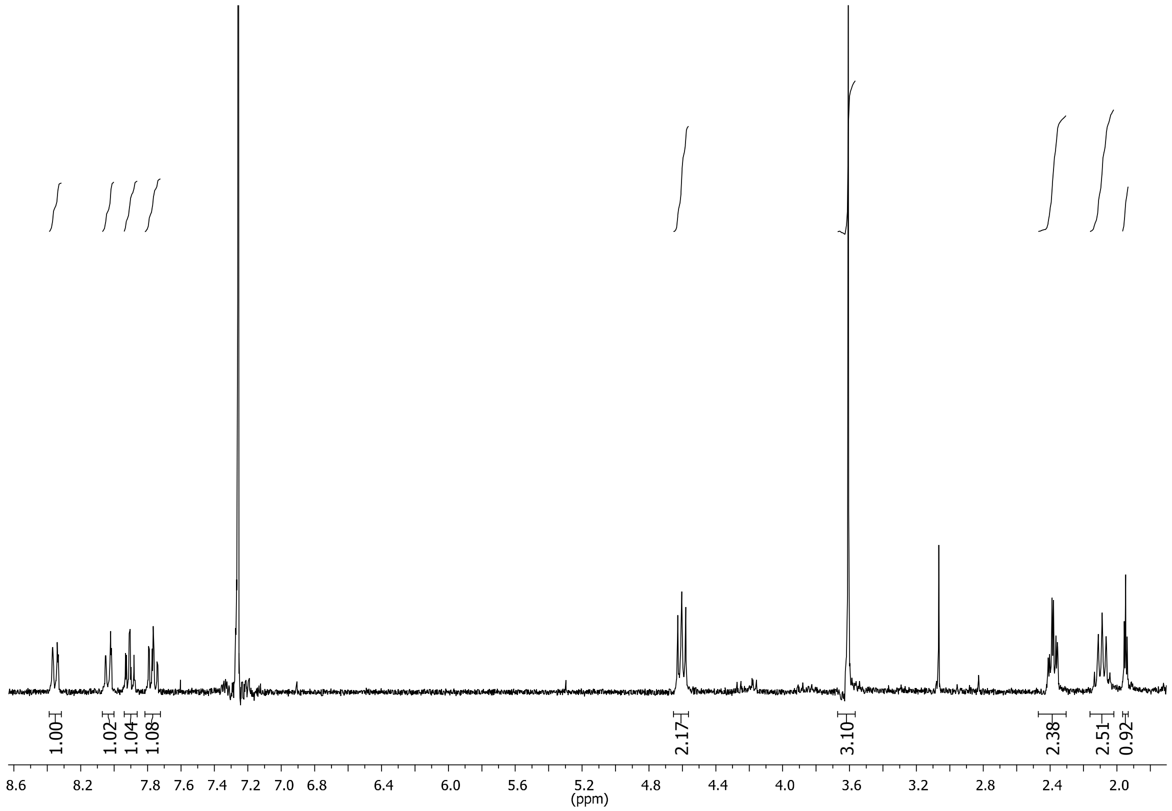

Figure S7. ^{1}H-NMR of compound **5c**.

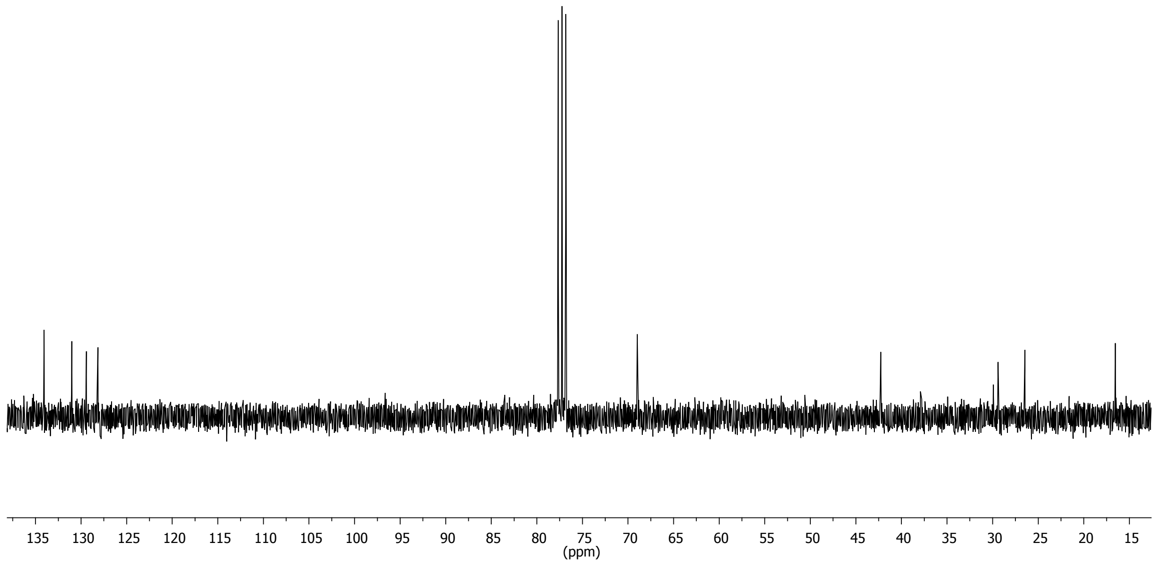

Figure S8. ^{13}C-NMR of compound **5c**.

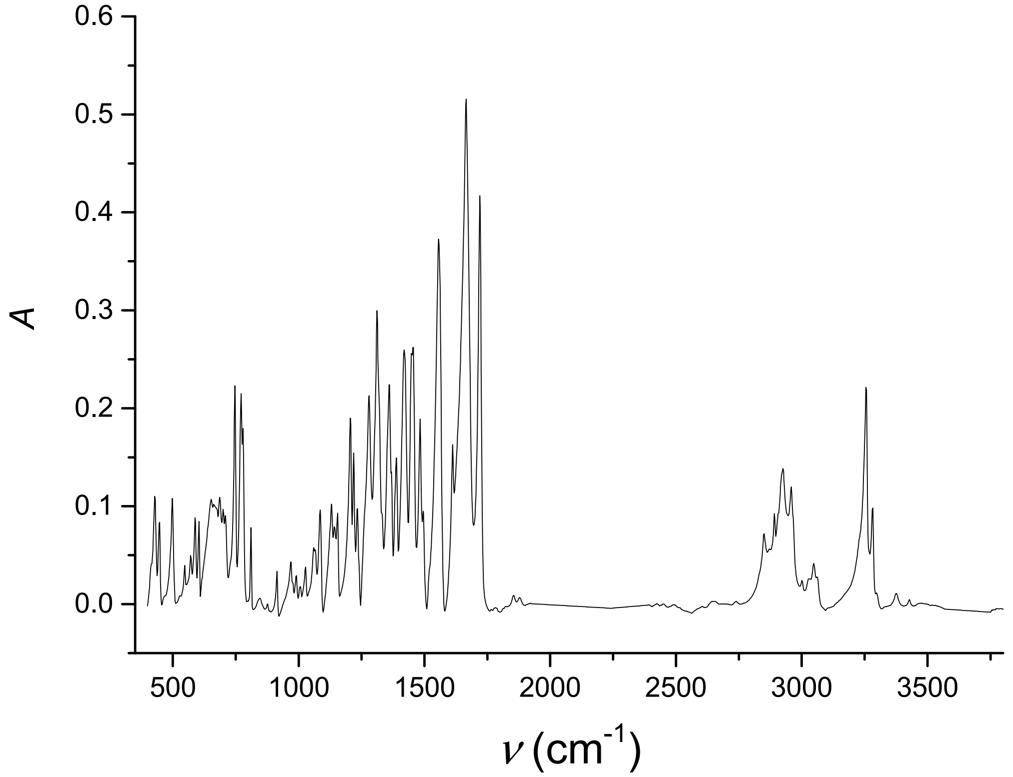

Figure S9. IR of compound **5c**.

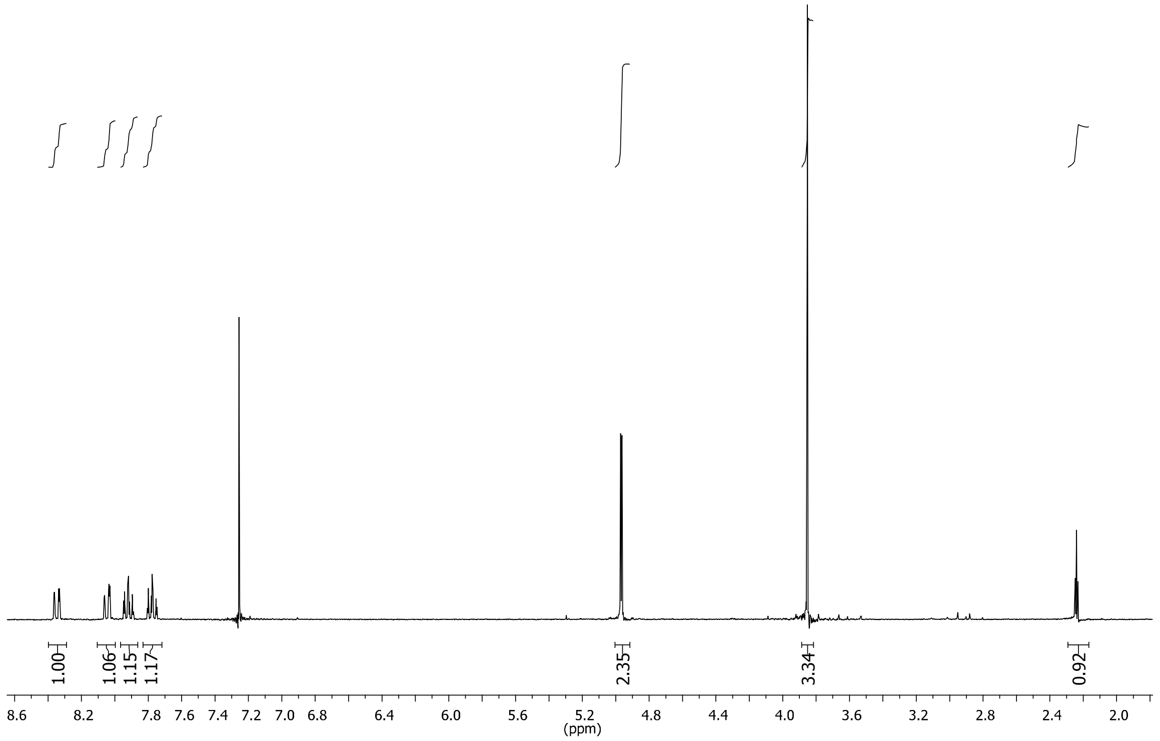

Figure S10. ^{1}H NMR of compound **6**.

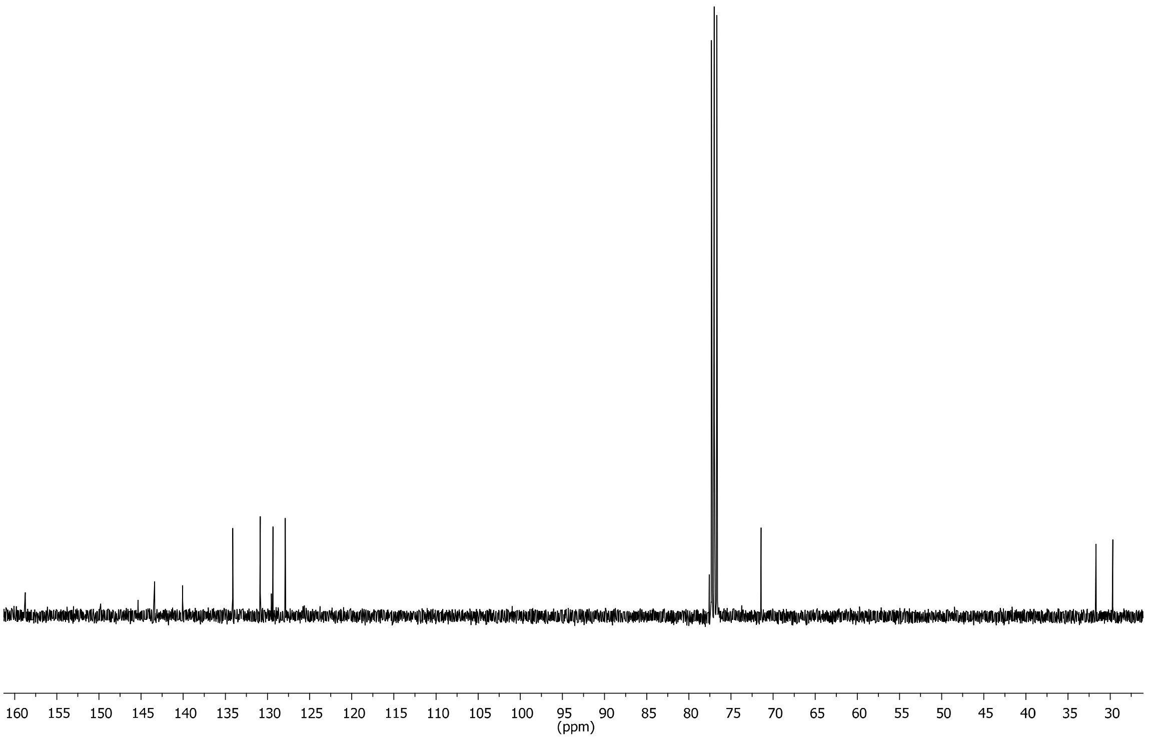

Figure S11. ^{13}C-NMR of compound **6**.

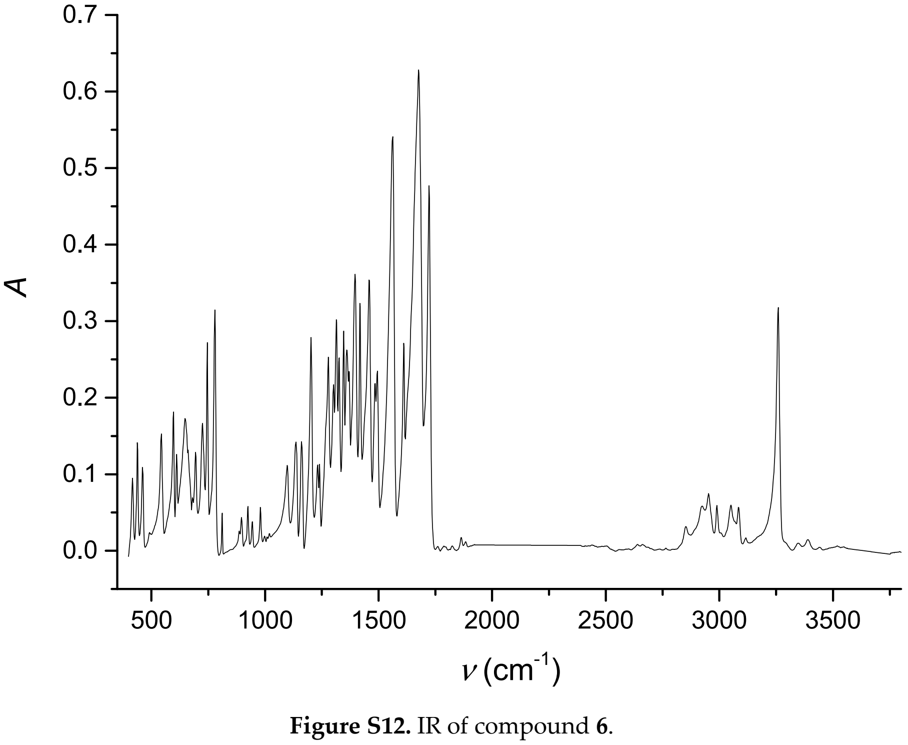

Figure S12. IR of compound **6**.

2. Model Experiments for Click-Chemistry with Flavins

3-Methyl-1-[(1-p-tolyl-1H-1,2,3-triazol-4-yl)methyl]alloxazine (**S1**): Under nitrogen atmosphere 3-methyl-1-propargylalloxazine (75 mg, 0.28 mmol), diisopropylethylamine (73 mg, 0.56 mmol), copper(I) iodide (3 mg, 0.01 mmol) and *p*-tolylazide (45 mg, 0.34 mmol) were mixed in 4 mL of dry *N*,*N*-dimethylformamide. After stirring for 24 h the solvent was evaporated and solid residue was dissolved in chloroform (10 mL) and washed with water (3 × 5 mL). Organic layer was dried with sodium sulfate. Sulfate was filtered off and solvent was evaporated *in vacuo*. Product **S1** (80 mg, 71%) was obtained as a solid. M.p. 278–281 °C. ^{1}H-NMR (300 MHz, $CDCl_3$) δ 8.36 (dd, *J* = 8.5, 0.9 Hz, 1H), 8.12 (d, *J* = 7.5 Hz, 1H), 7.92 (ddd, *J* = 8.5, 5.2, 1.5 Hz, 1H), 7.78 (ddd, *J* = 8.4, 6.9, 1.4 Hz, 1H), 7.56 (s, *J* = 8.5 Hz, 1H), 7.29 (dd, *J* = 8.6, 2.7 Hz, 2H), 7.25 (m, 2H), 5.90 (s, 2H), 3.62 (s, 3H), 2.40 (s, 3H). ^{13}C-NMR (75 MHz, $CDCl_3$) δ 159.85, 150.60, 144.63, 144.22, 143.42, 140.43, 139.10, 134.73, 134.18, 130.89, 130.32, 129.54, 128.19, 121.84, 120.61, 118.94, 71.63, 37.58, 32.12, 29.38, 21.23. HRMS-ESI$^+$ *m*/*z*: $[M + H]^+$ calcd for $C_{21}H_{18}N_7O_2$ 400.1517, found 400.1517.

1-[(1-Benzyl)-1H-1,2,3-triazol-4-yl)methyl]-3-methylalloxazine (**S2**): Under nitrogen atmosphere 3-methyl-1-propargylalloxazine (75 mg, 0.28 mmol), diisopropylethylamine (73 mg, 0.56 mmol), copper(I) iodide (3 mg, 0.01 mmol) and benzylazide (45 mg, 0.34 mmol) were mixed in 4 mL of dry *N*,*N*-dimethylformamide. After stirring for 24 h the solvent was evaporated and solid residue was dissolved in chloroform (10 mL) and washed with water (3 × 5 mL). Organic layer was dried with sodium sulfate. Sulfate was filtered off and solvent was evaporated *in vacuo*. Product **S2** (70 mg, 62%) was obtained as a solid. M.p. 275–277 °C. ^{1}H-NMR (300 MHz, $CDCl_3$) δ 8.34 (d, *J* = 8.8 Hz, 1H), 8.06 (d, *J* = 8.9 Hz, 1H), 7.89 (t, *J* = 7.6 Hz, 1H), 7.81–7.72 (m, 1H), 7.60 (s, 1H), 7.37–7.31 (m, 3H), 7.24–7.19 (m, 2H), 5.78 (s, 2H), 5.47 (s, 2H), 3.60 (s, 3H). ^{13}C-NMR (101 MHz, $CDCl_3$) δ 159.81, 150.58, 146.63, 143.20, 140.42, 134.49, 133.90, 130.74, 129.65, 129.33, 129.07, 128.76, 128.10, 128.03, 54.35, 29.21. HRMS-ESI$^+$ *m*/*z*: $[M + H]^+$ calcd for $C_{21}H_{18}N_7O_2$ 400.1522, found 400.1519.

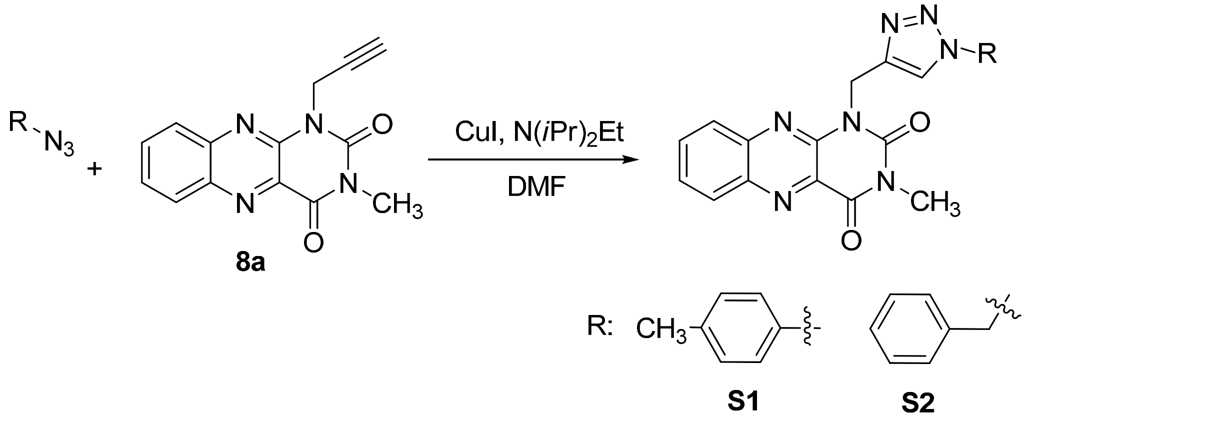

Scheme S1. Model experiments for click chemistry.

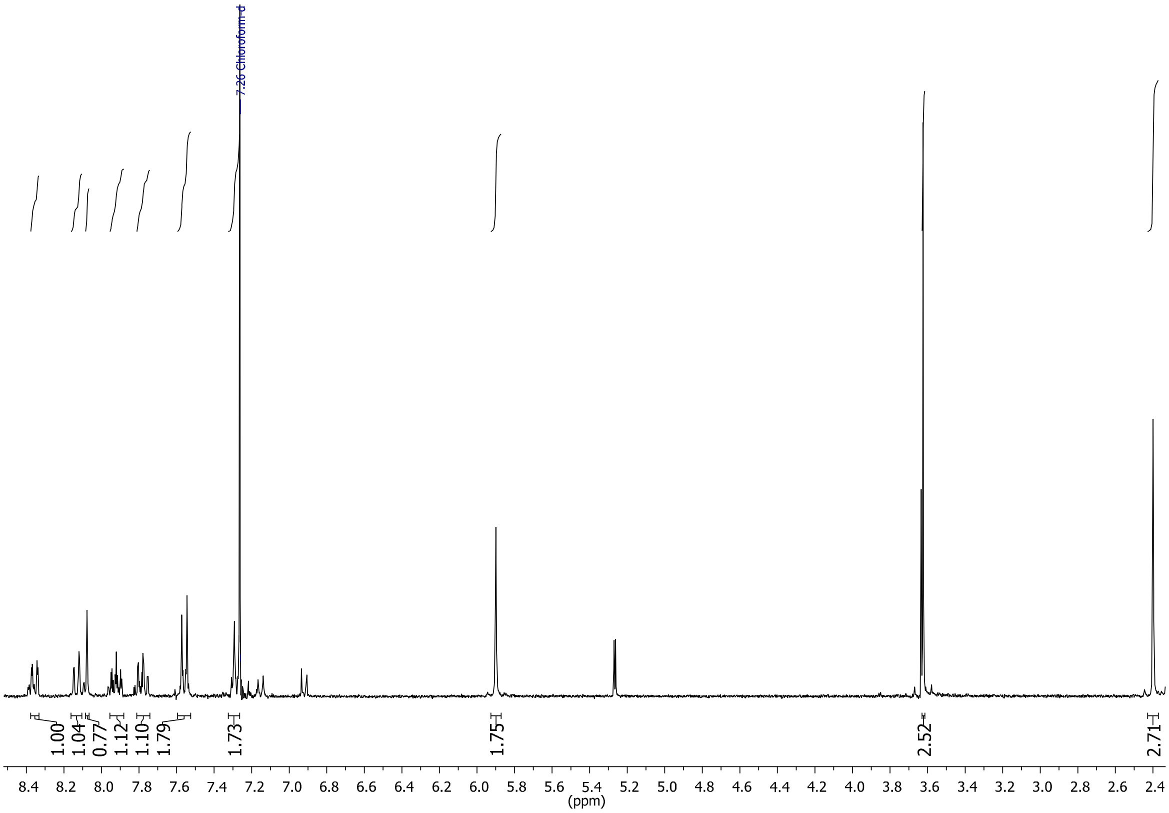

Figure S13. ^{1}H-NMR of compound **S1**.

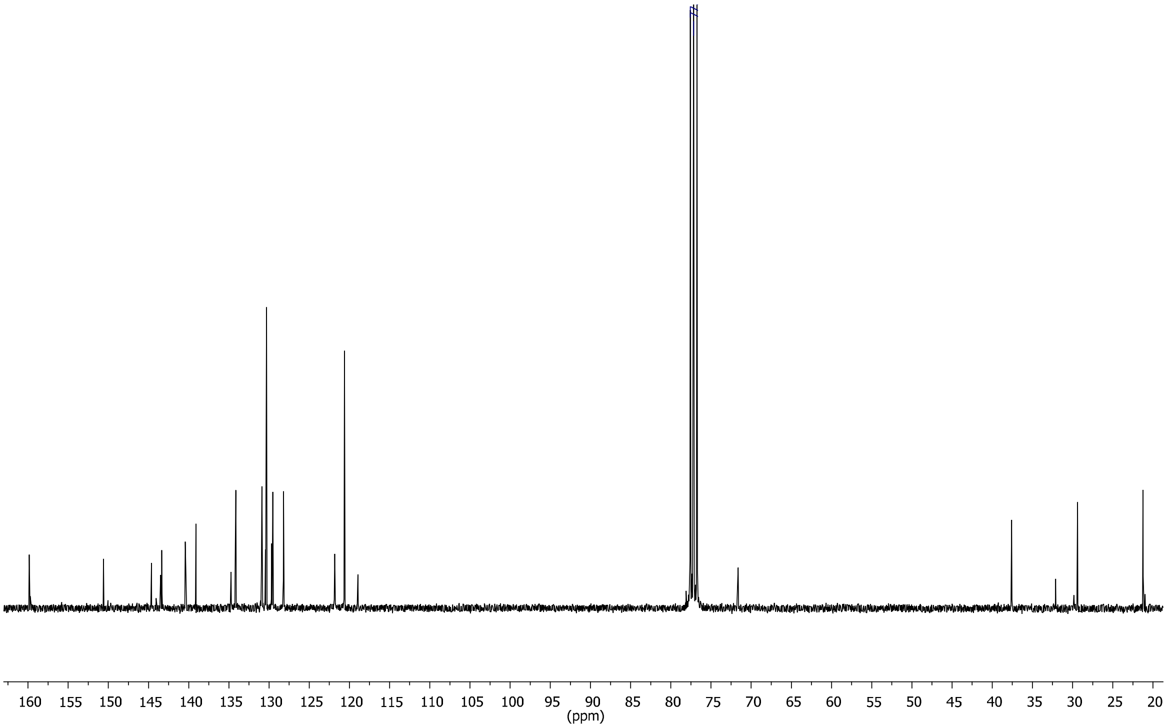

Figure S14. ^{13}C-NMR of compound **S1**.

3. Characterization of Flavin-Cyclodextrin Conjugates 8 and 9

The NMR spectra were measured on Bruker AVANCE-600 instrument (^{1}H at 600.13 MHz and ^{13}C at 150.9 MHz) with a cryo-probe in D2O at 40 °C. Homonuclear 2D-NMR spectra (H,H-COSY) and heteronuclear 2D-NMR spectra (H,C-HSQC and H,C-HMBC) were used for structural assignment of proton and carbon signals of compounds **8** and **9**.

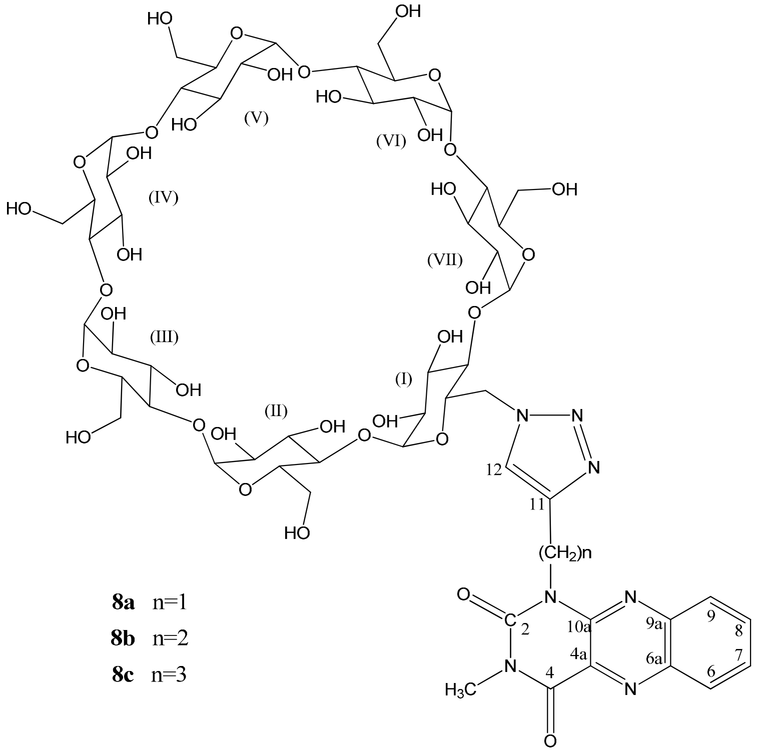

8a n=1
8b n=2
8c n=3

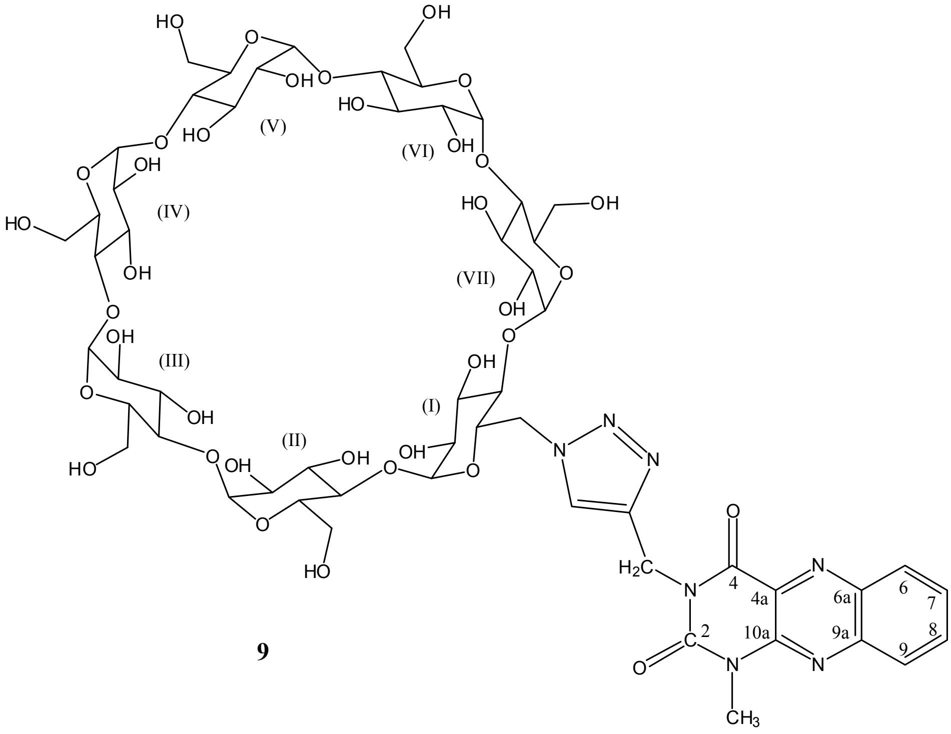

9

Table S1. Carbon NMR data of compounds **8** and **9** (aglycone part) in D_2O at 40 °C.

Compound	C-2	C-4	C-4a	C-6a	C-6	C-7	C-8	C-9	C-9a	C-10a	N-CH3	C-11	C-12	-(CH2)n-
**8a**	153.86	163.60	132.89	142.01	132.40	132.80	137.33	129.88	145.22	147.20	31.70	145.76	128.87	40.26
**8b**	154.13	163.42	132.92	141.92	132.42	132.74	137.24	129.40	144.74	?	31.60	148.24	125.51	44.61; 26.18
**8c**	154.05	163.34	132.72	141.85	132.47	132.71	137.28	129.46	145.21	147.74	31.56	150.76	124.97	44.25; 27.60; 24.41
**9**	153.56	163.03	133.00	141.71	132.19	132.31	137.02	129.85	145.43	148.27	32.14	144.38	129.14	39.51

Table S2. Proton NMR data of compounds **8** and **9** (aglycone part) in D_2O at 40 °C.

Compound	H-6	H-7	H-8	H-9	N-CH3	H-12	-(CH2)n-
**8a**	8.33 dd (8.6; 1.4)	7.98 ddd (8.6; 6.7; 1.5)	8.10 ddd (8.6; 6.7; 1.4)	8.28 dd (8.6; 1.5)	3.52 s	7.88 s	5.76 d + 5.66 d (15.8)
**8b**	8.35 dd (8.4; 1.4)	8.05 ddd (8.4; 6.9; 1.4)	8.13 ddd (8.4; 6.9; 1.4)	7.90 dd (8.4; 1.4)	3.57 s	7.59 s	5.08 m + 4.56 m<br>3.37 m + 3.30 m
**8c**	8.33 dd (8.5; 1.4)	8.03 ddd (8.5; 6.9; 1.4)	8.13 ddd (8.5; 6.9; 1.4)	7.95 dd (8.5; 1.4)	3.53 s	7.55 s	4.60 m + 4.45 m<br>2.26 m (2H)<br>2.87 m (2H)
**9**	8.26 d (8.5)	7.91 ddd (8.5; 6.5; 1.5)	8.04 bt (8.5; 6.5)	8.06 bdd (8.5; 1.5)	3.74 s	7.83 s	5.6 d + 5.18 d (15.1)

Table S3. Carbon NMR data of compounds **8** and **9** (β-CD part) in D_2O at 40 °C.

Compound	Residue	C-1′	C-2′ + C-3′ + C-5′ (not Assigned)			C-4′	C-6′
	I	103.89	75.96	74.88	74.93	85.21	53.87
	II	104.70	75.85 (3)	74.84	74.51 (2)	83.14	61.77
	III	104.60	75.73	74.63 (2)	74.41	83.79	62.72–63.19
**8a**	IV	104.02	75.58	74.58 (3)	74.30	84.02	62.72–63.19
	V	104.62	75.55		74.28	83.17	62.72–63.19
	VI	104.60			74.26	83.70	62.72–63.19
	VII	104.30				84.02	62.72–63.19
	I	103.93	76.34	75.34	74.63	85.52	53.60
	II	105.05	76.03	75.22	74.55	82.96	62.74
	III	104.78	75.80	75.04	74.52	83.70	62.74
**8b**	IV	103.48	75.70	74.82	74.34 (2)	84.06	62.92
	V	105.00	75.65	74.75	74.13	82.27	62.46
	VI	104.78	75.60	74.73	74.07	84.15	63.27
	VII	104.53	75.49	74.71		83.70	62.42
	I	104.28	75.99	75.06	74.50	85.60	53.64
	II	104.47	75.91	74.83	74.47	82.96	62.62–63.18
	III	104.49	75.82	74.71	74.46	83.49	62.62–63.18
**8c**	IV	104.71	75.81	74.64	74.41	83.88	61.61
	V	104.03	75.70	74.62	74.28 (2)	83.64	62.62
	VI	104.64	75.63	74.61	74.15	82.80	62.62–63.18
	VII	104.64	75.34	74.58		83.86	62.62–63.18
	I	104.92	75.94	74.88	74.42 (2)	86.04	53.47
	II	104.74	75.90	74.84	74.40 (2)	84.09	61.57
	III	104.68	75.74 (2)	74.78	74.27	83.70	62.71
**9**	IV	104.54	75.68	74.68 (2)	74.07	83.57	62.76
	V	104.42	75.53	74.66	72.83	83.52	62.78
	VI	103.96	75.44	74.46		83.14	62.82
	VII	103.92				82.99	63.36

Table S4. Proton NMR data of compounds **8** and **9** (β-CD part) in D_2O at 40 °C.

Compound		H-1′	H-2′	H-3′	H-4′	H-5′	H-6′a + H-6′b
	I	4.864	3.599	3.739	3.467	3.744	4.842; 4.519
	II	4.978	3.554	3.794	3.381	3.283	2.943; 2.667
	III	4.978	3.594	3.812	3.524	3.717	3.48–3.98
**8a**	IV	4.958	3.555	3.668	3.404	3.177	3.585; 3.413
	V	5.082	3.645	3.943	3.558	3.878	3.48–3.98
	VI	5.041	3.667	3.969	3.643	3.917	3.48–3.98
	VII	5.059	3.610	3.785	3.424	3.519	3.48–3.98
	I	5.070	3.678	3.797	3.428	3.418	4.718; 4.288
	II	5.112	3.691	4.090	3.715	3.910	3.67–3.95
	III	4.839	3.522	3.592	3.582	3.282	3.67–3.95
**8b**	IV	4.852	3.406	3.382	3.177	2.446	3.212; 3.081
	V	5.168	3.667	3.933	3.592	4.167	3.67–3.95
	VI	5.041	3.711	4.136	3.724	3.715	4.146; 4.028
	VII	4.986	3.598	3.650	3.411	2.839	3.466; 3.408

Table S4. *Cont.*

**Compound**		**H-1′**	**H-2′**	**H-3′**	**H-4′**	**H-5′**	**H-6′a + H-6′b**
	I	5.007	3.721	3.964	3.600	3.874	4.827; 4.498
	II	5.015	3.626	3.882	3.558	3.697	3.53–3.86
	III	4.970	3.598	3.843	3.478	3.406	3.53–3.86
**8c**	IV	4.951	3.556	3.750	3.559	3.338	3.237; 2.974
	V	4.933	3.517	3.668	3.406	3.043	3.434; 3.300
	VI	5.040	3.601	3.806	3.565	3.751	3.53–3.86
	VII	5.160	3.699	3.930	3.510	~3.75	3.53–3.86
	I	5.104	3.700	4.035	3.618	4.099	3.988 + 3.935
	II	5.091	3.650	3.939	3.583	4.059	3.75–3.91
	III	5.085	3.666	3.747	3.609	4.001	3.75–3.91
**9**	IV	4.957	3.592	3.762	3.574	3.900	3.75–3.91
	V	4.945	3.509	3.726	3.350	3.254	2.970 + 2.367
	VI	4.937	3.518	3.599	3.362	3.067	3.518 + 3.443
	VII	4.827	3.569	3.642	3.386	3.457	4.673 + 4.301

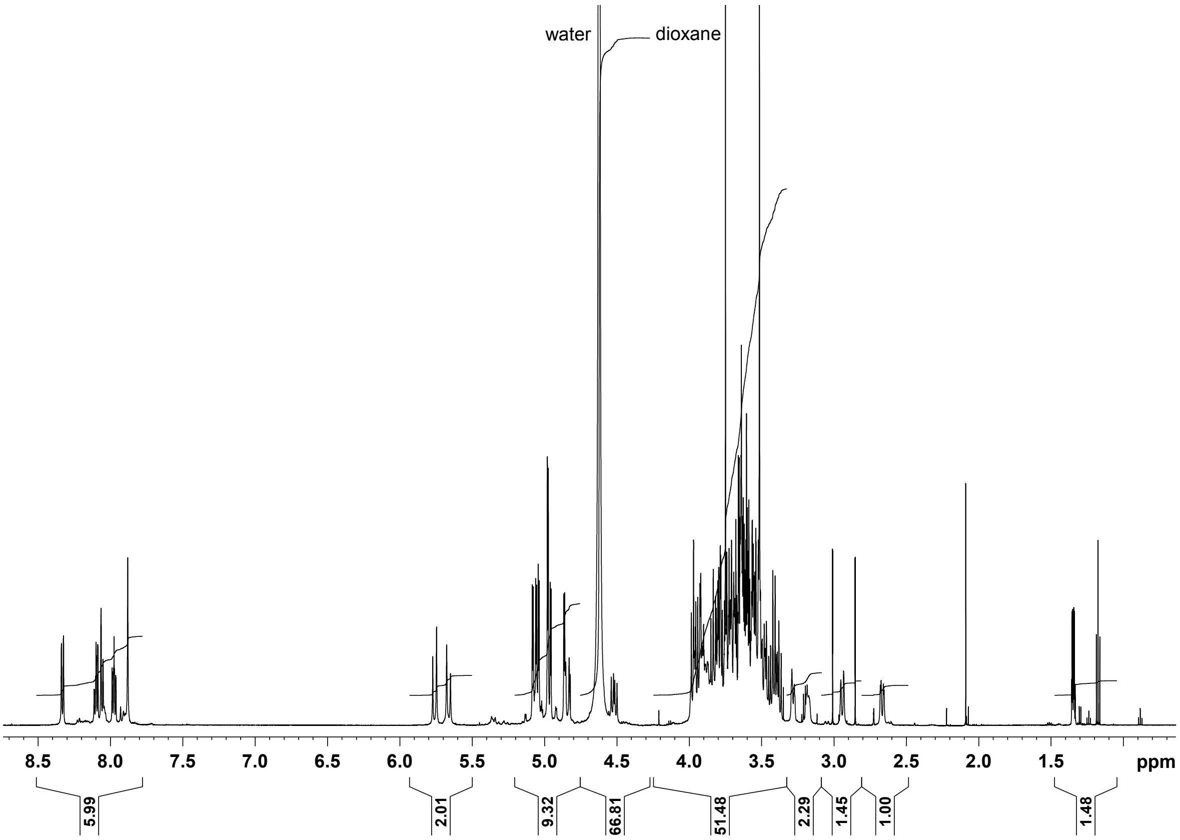

Figure S15. ^{1}H-NMR of compound **8a**.

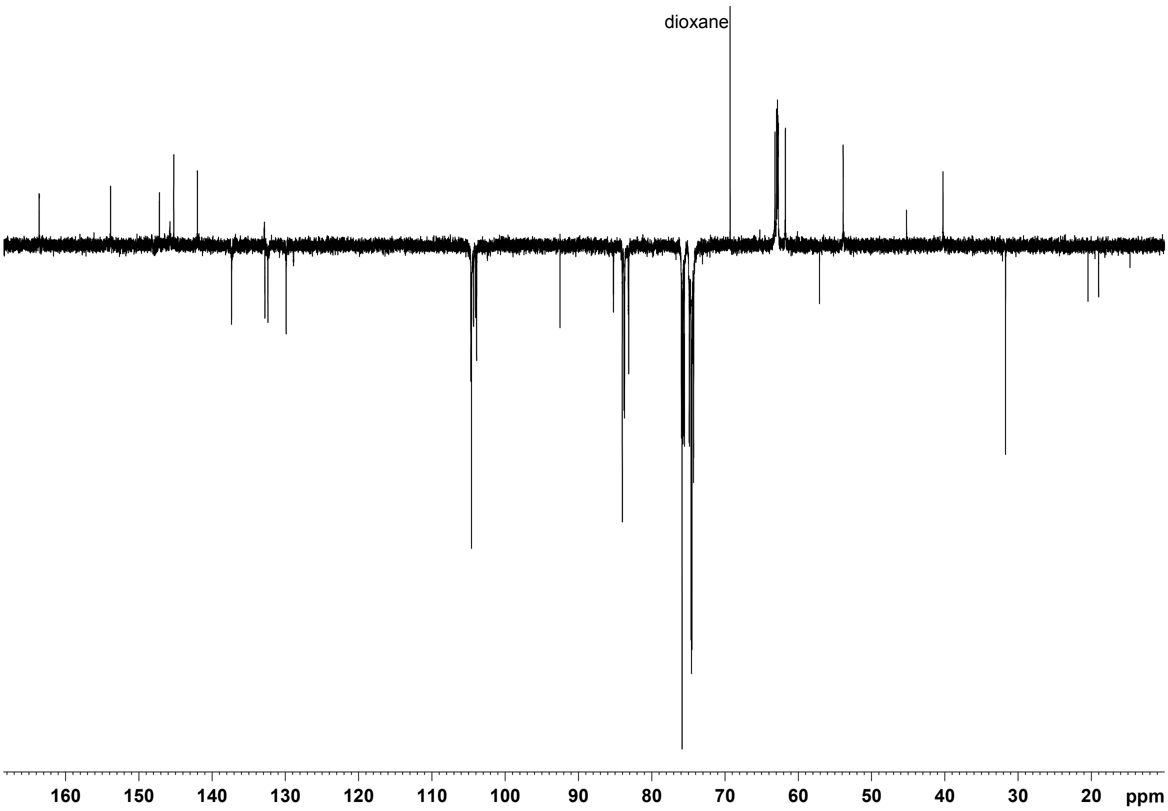

Figure S16. ^{13}C-NMR of compound **8a**.

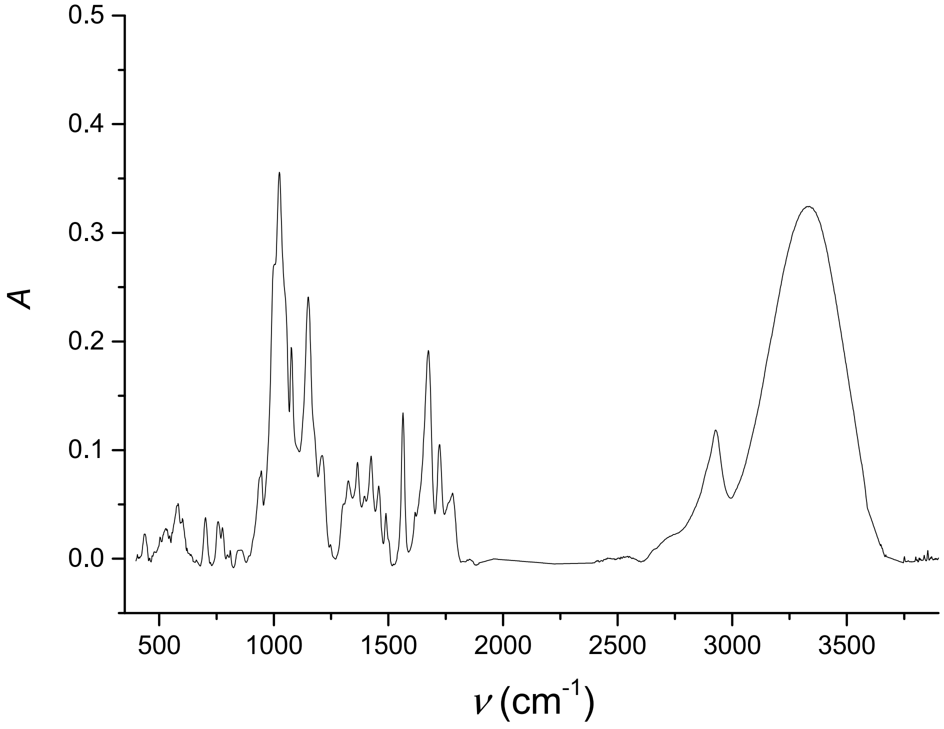

Figure S17. IR of compound **8a**.

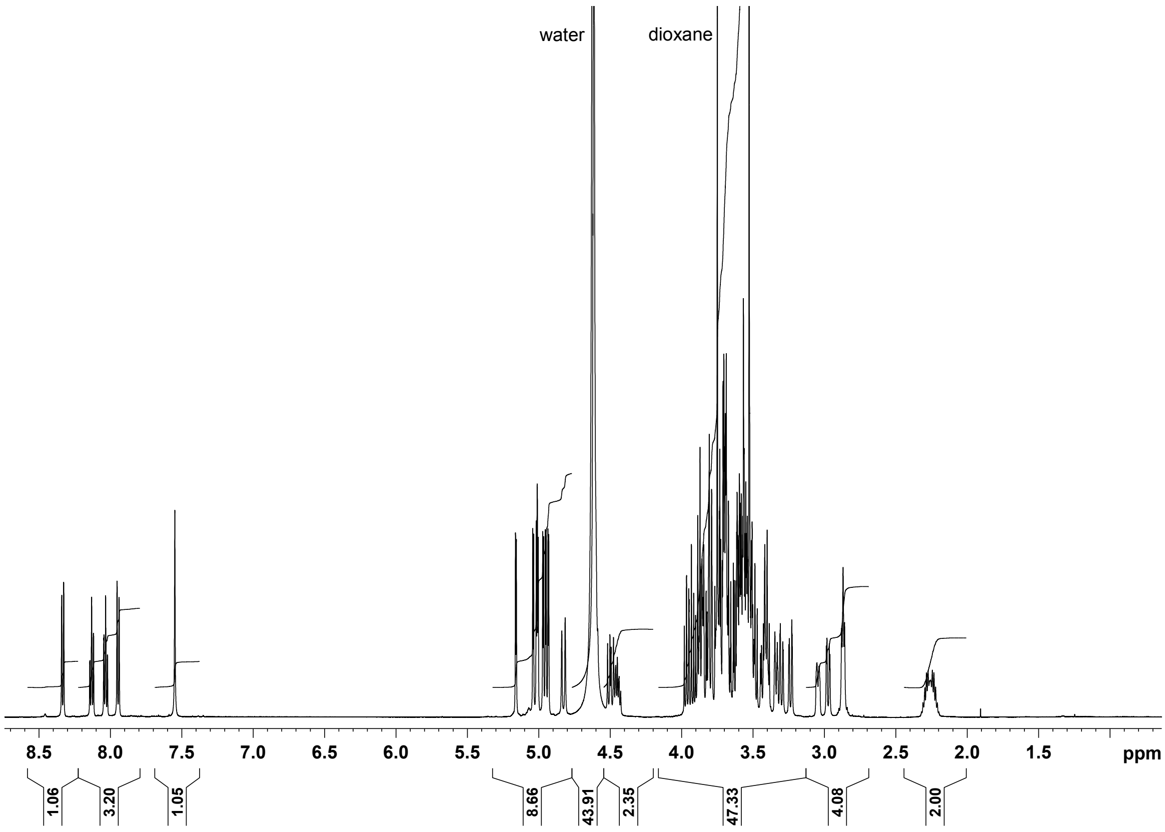

Figure S18. ^{1}H-NMR of compound **8b**.

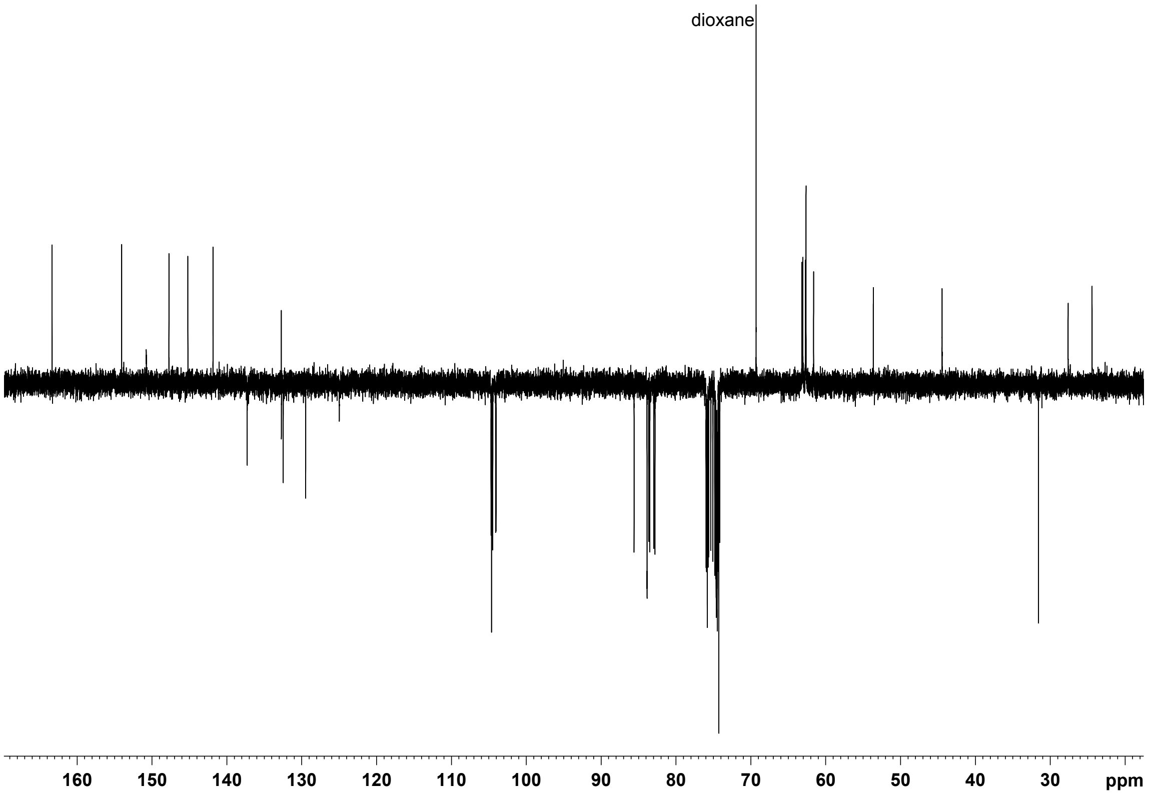

Figure S19. ^{13}C-NMR of compound **8b**.

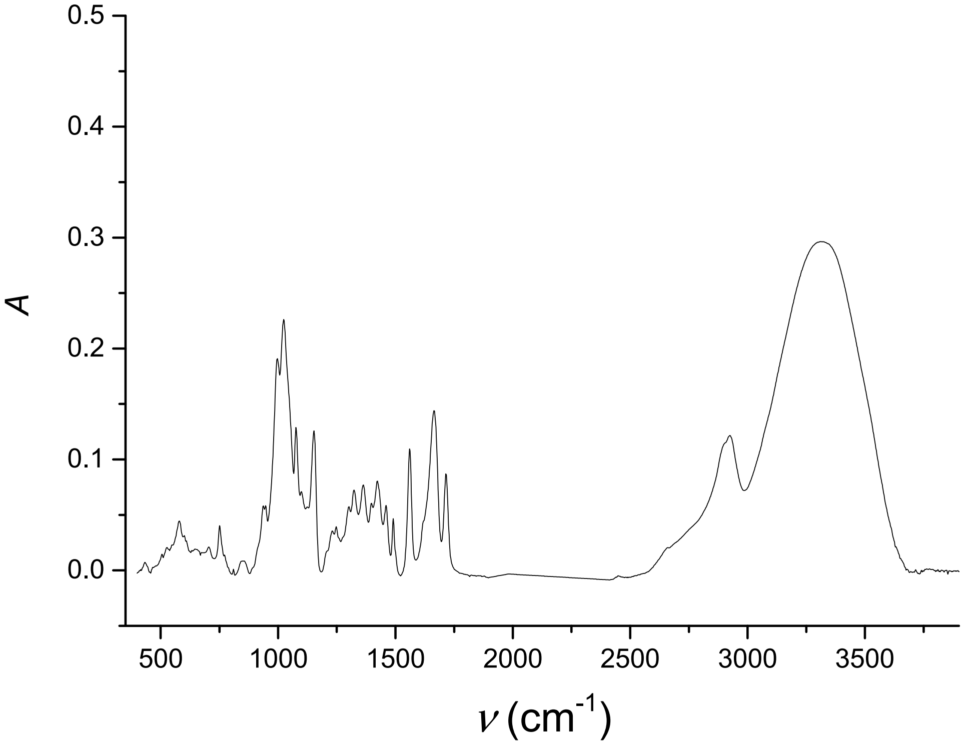

Figure S20. IR of compound **8b**.

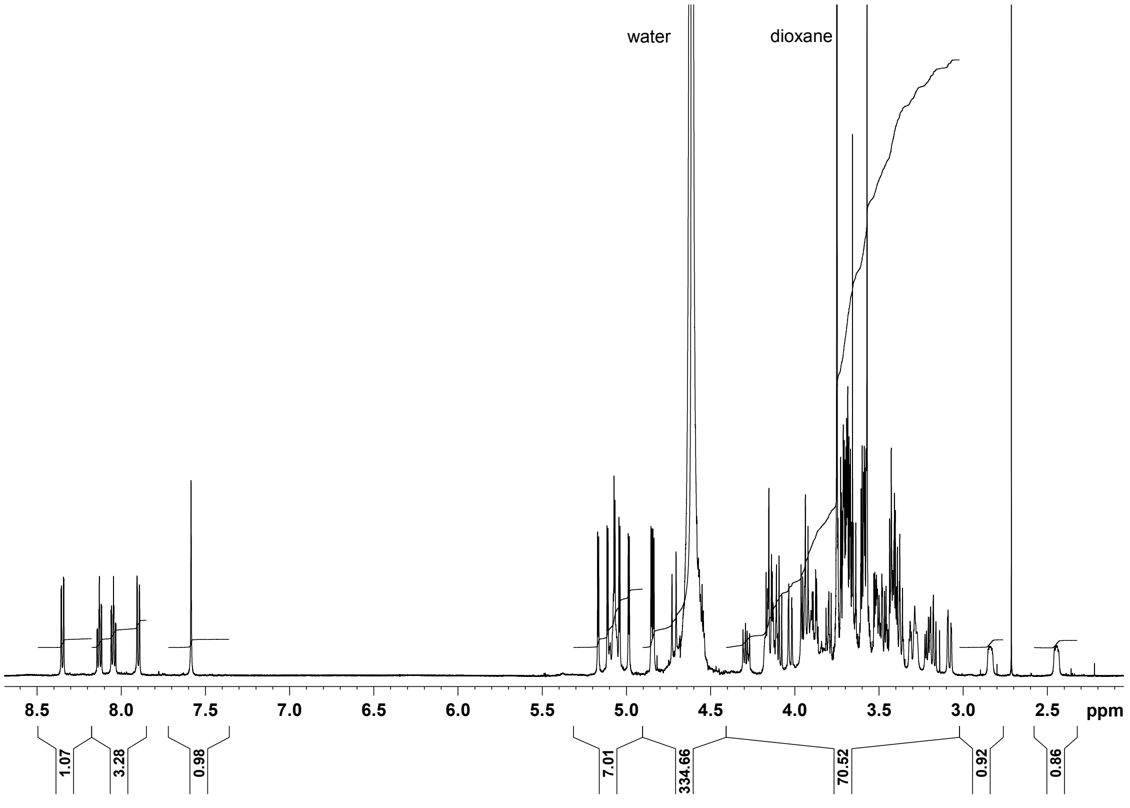

Figure S21. ^{1}H-NMR of compound **8c**.

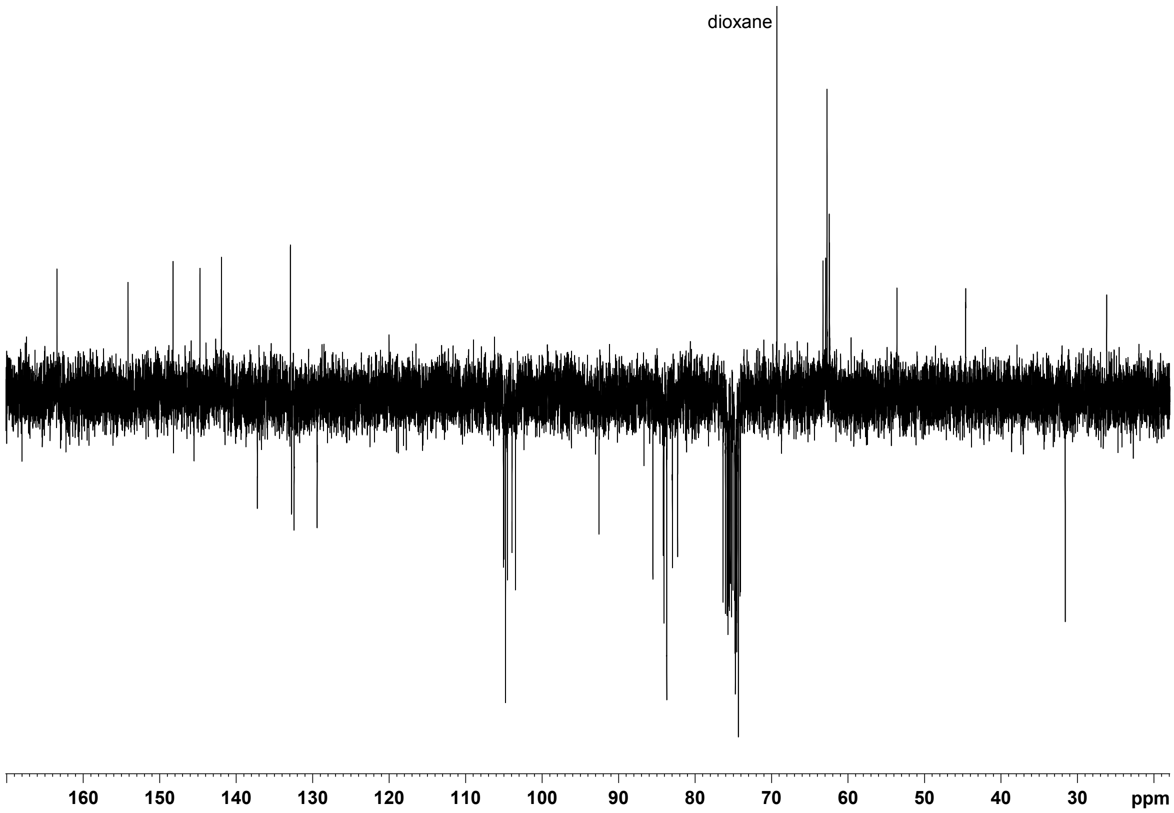

Figure S22. ^{13}C-NMR of compound **8c**.

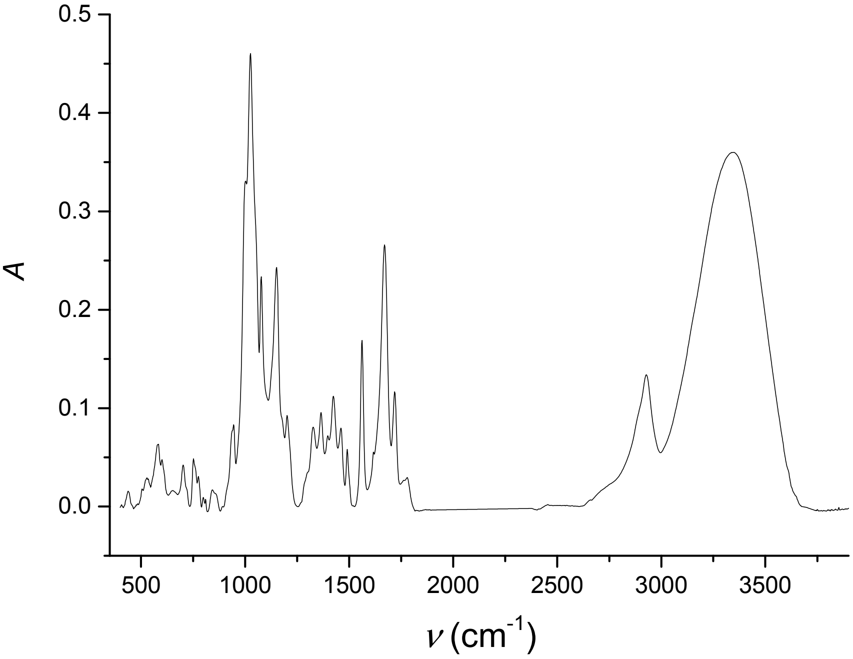

Figure S23. IR of compound **8c**.

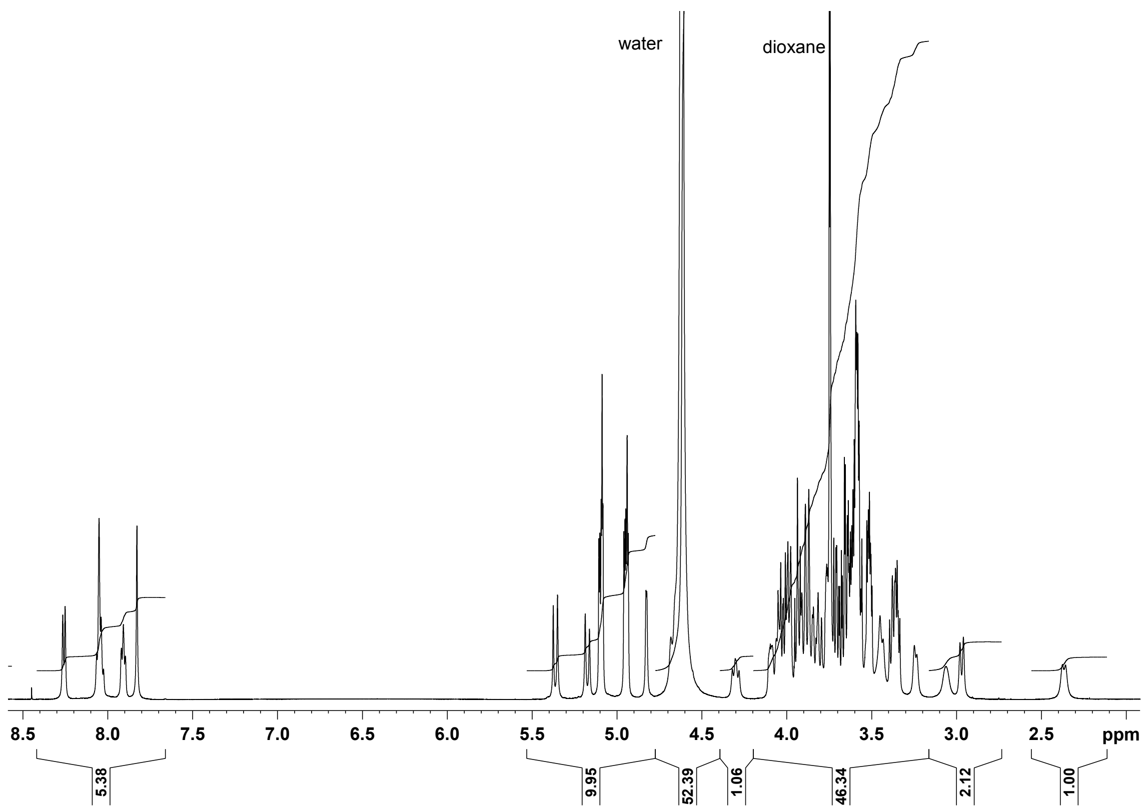

Figure S24. ^{1}H-NMR of compound **9**.

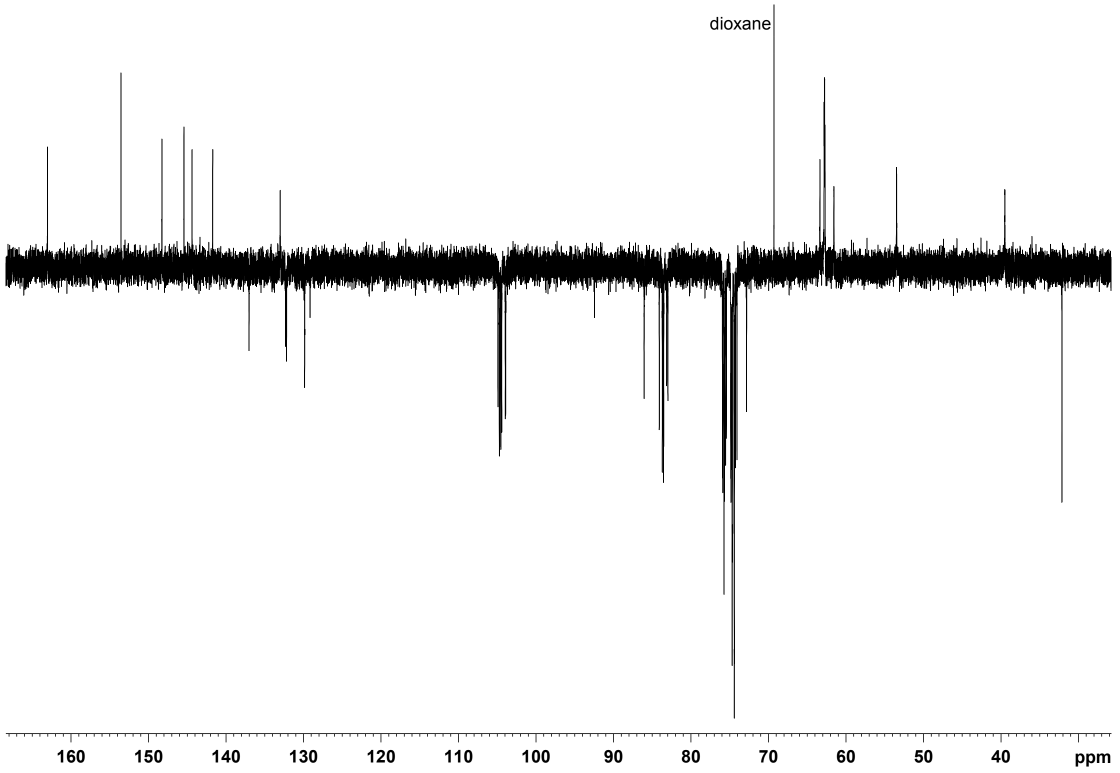

Figure S25. ^{13}C-NMR of compound **9**.

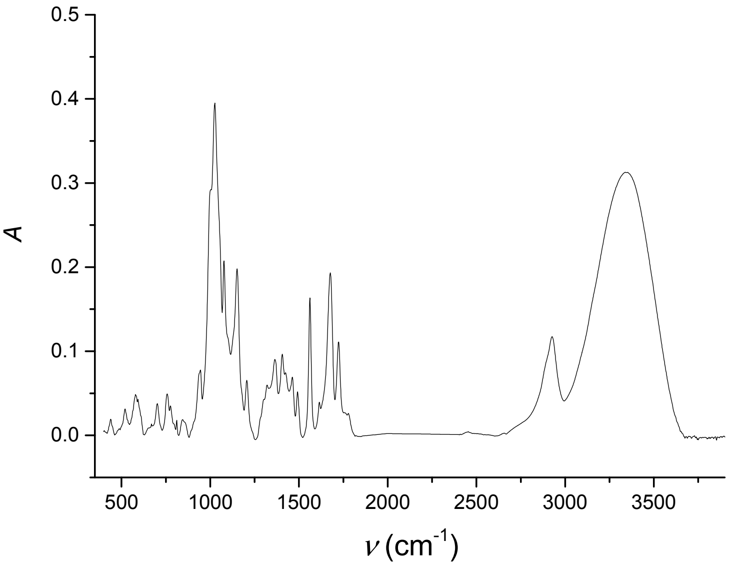

Figure S26. IR of compound **9**.

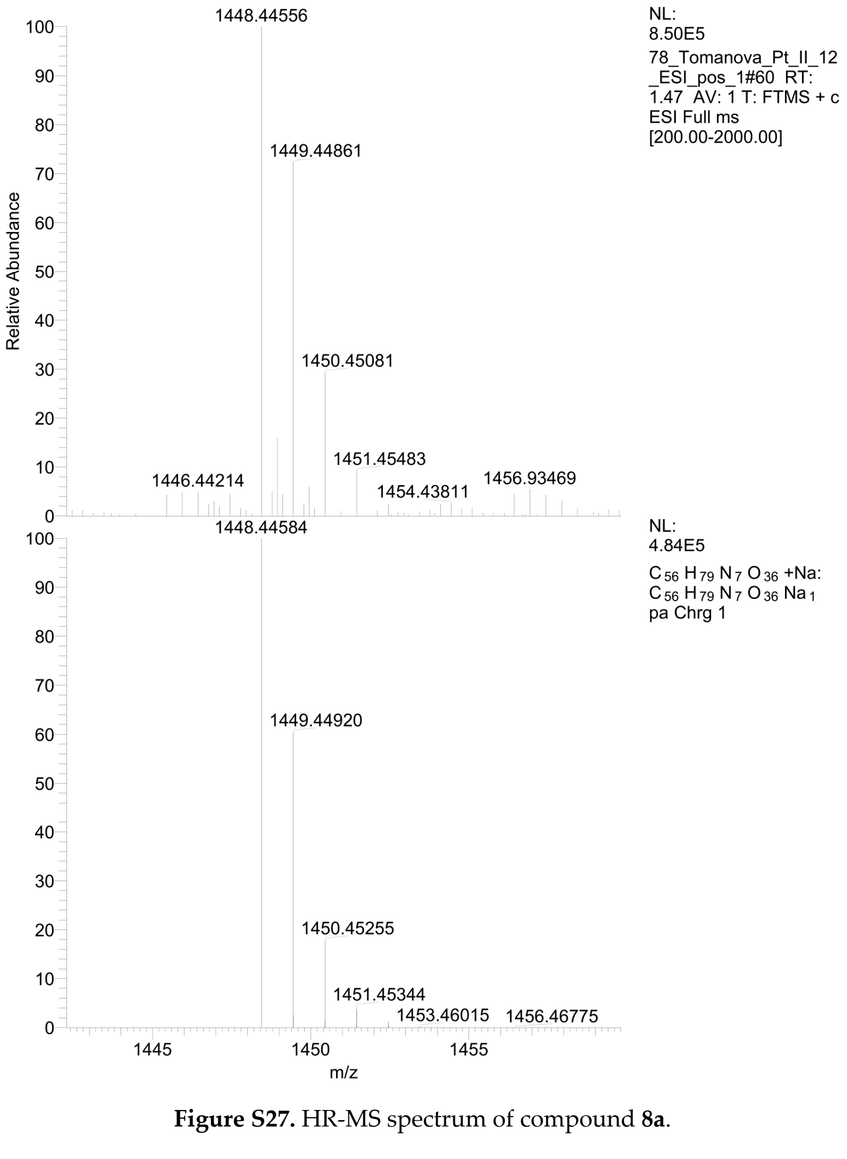

Figure S27. HR-MS spectrum of compound **8a**.

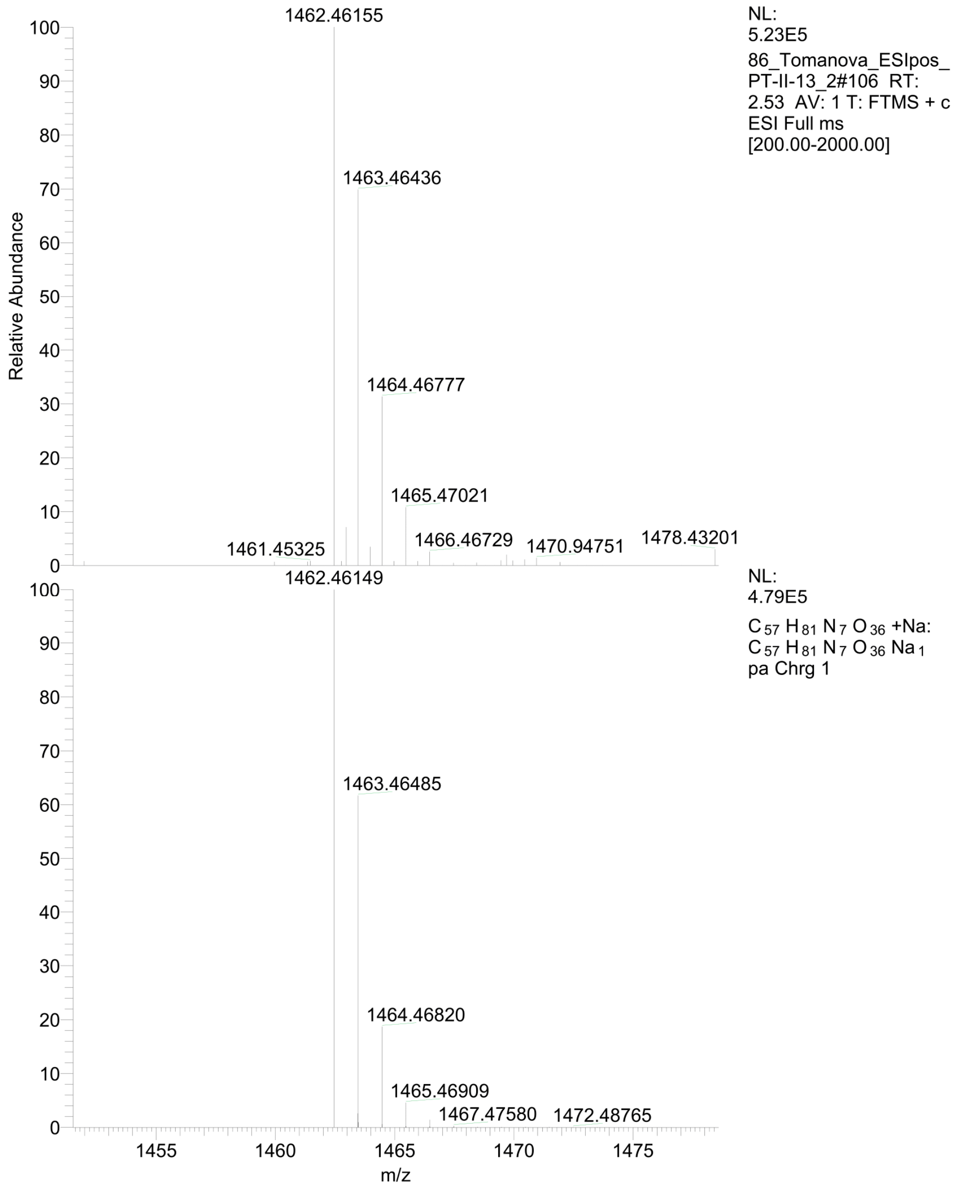

Figure S28. HR-MS spectrum of compound **8b**.

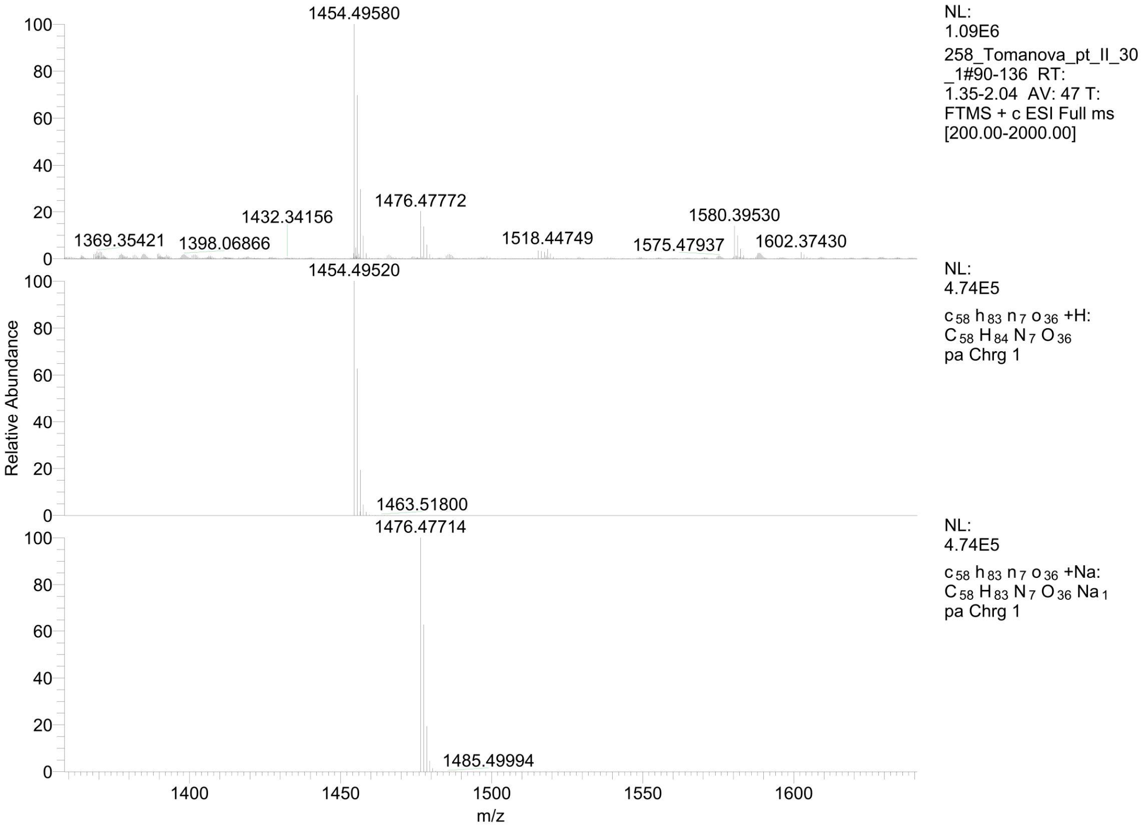

Figure S29. HR-MS spectrum of compound **8c**.

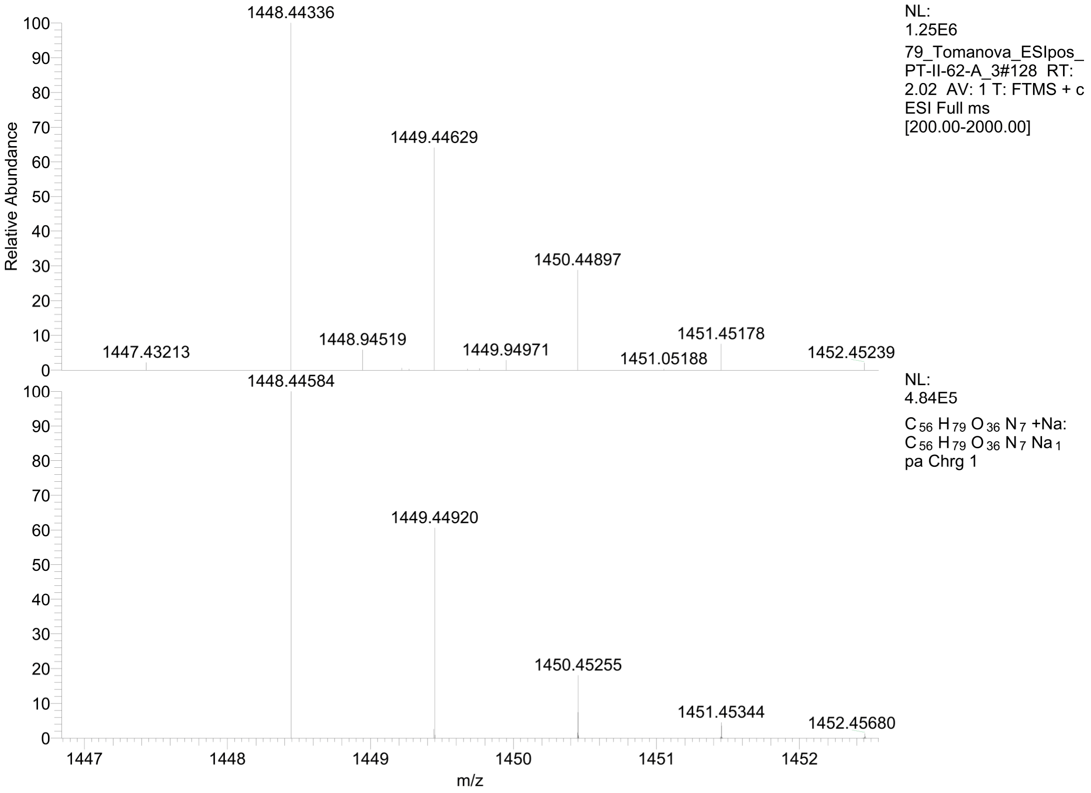

Figure S30. HR-MS spectrum of compound **9**.

4. Characterization of Flavinium Catalysts 3 and 4

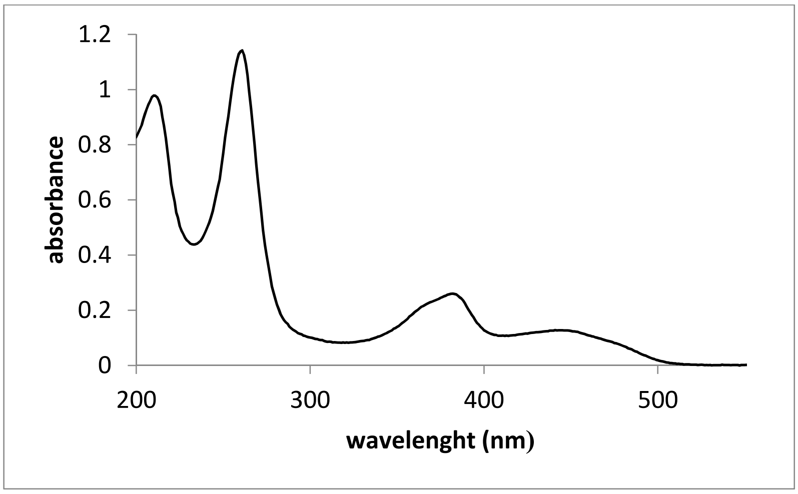

Figure S31. UV-VIS spectrum of compound **3a**.

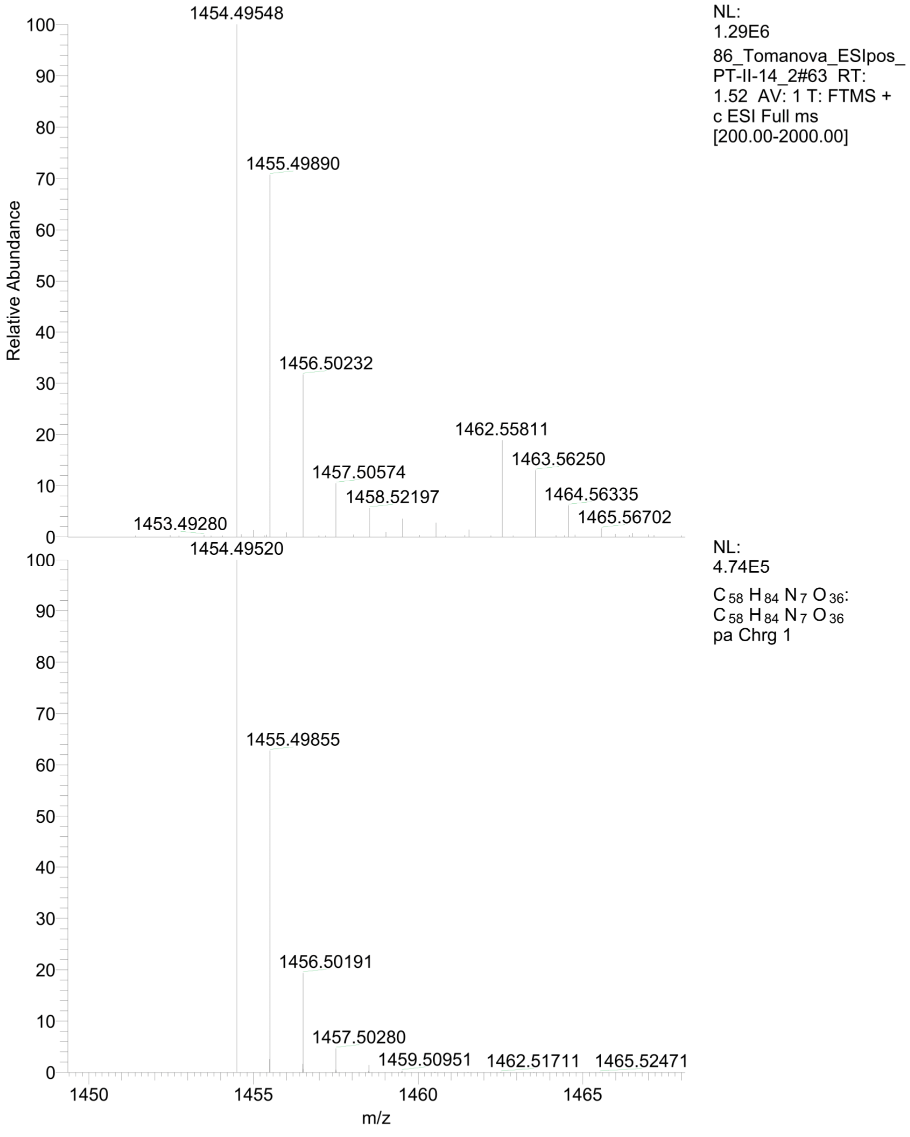

Figure S32. HR-MS spectrum of compound **3a**.

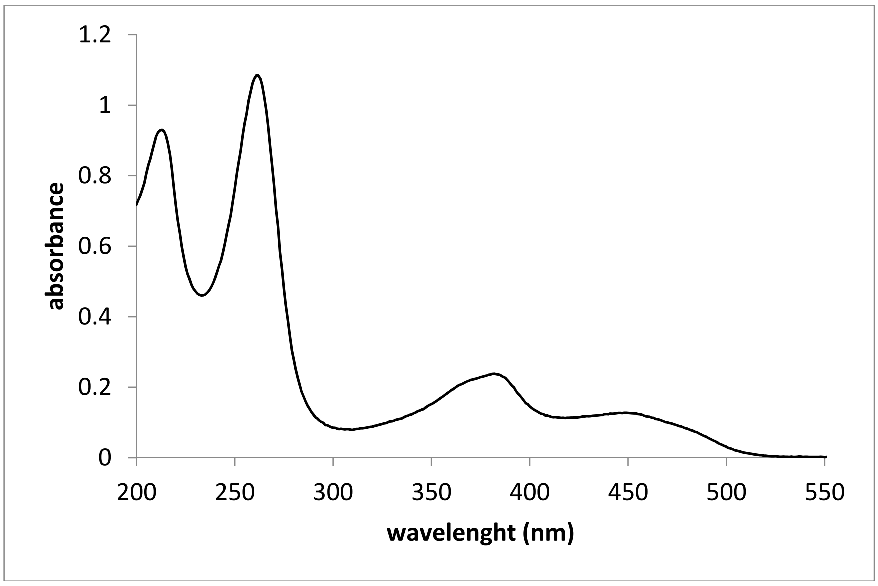

Figure S33. UV-VIS spectrum of compound **3b**.

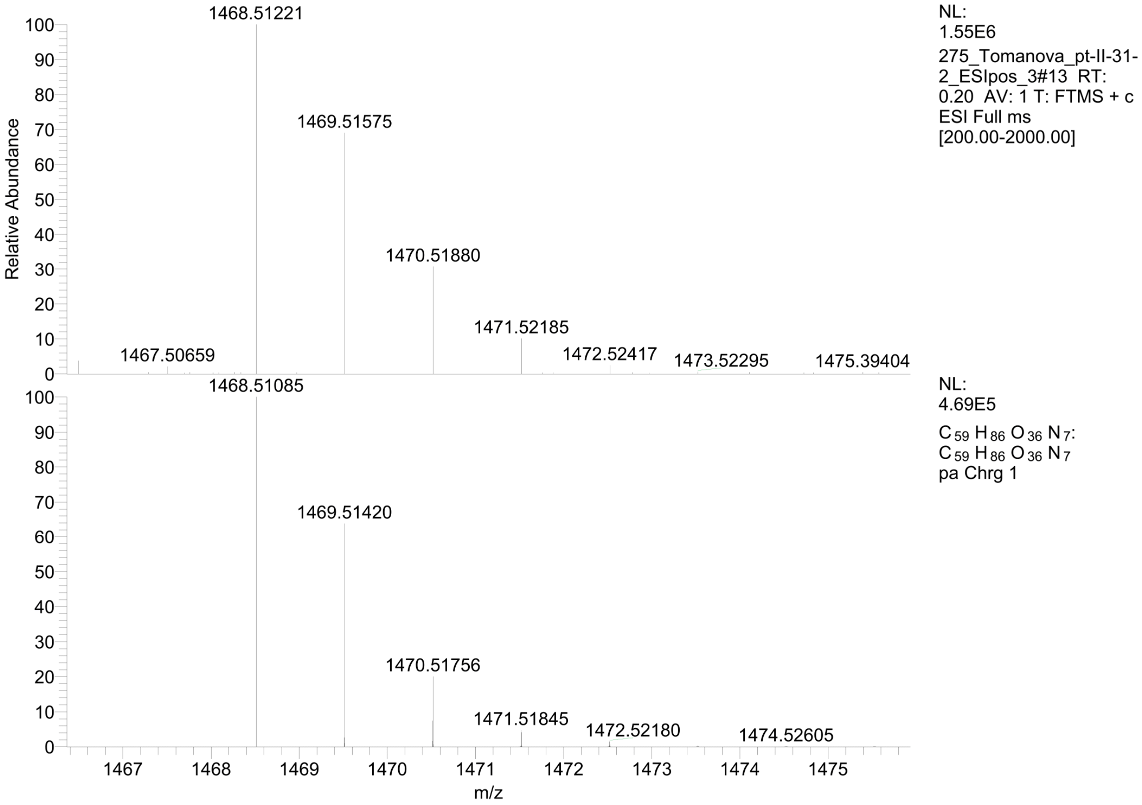

Figure S34. HR-MS spectrum of compound **3b**.

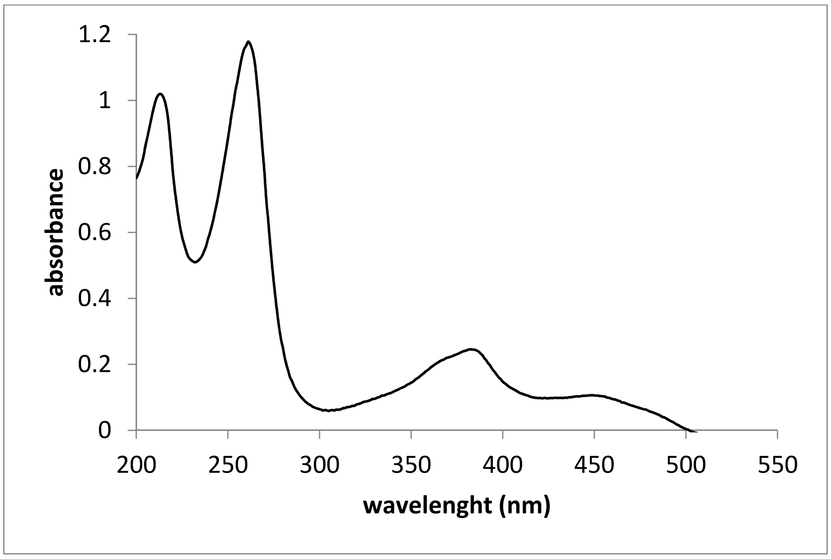

Figure S35. UV-VIS spectrum of compound **3c**.

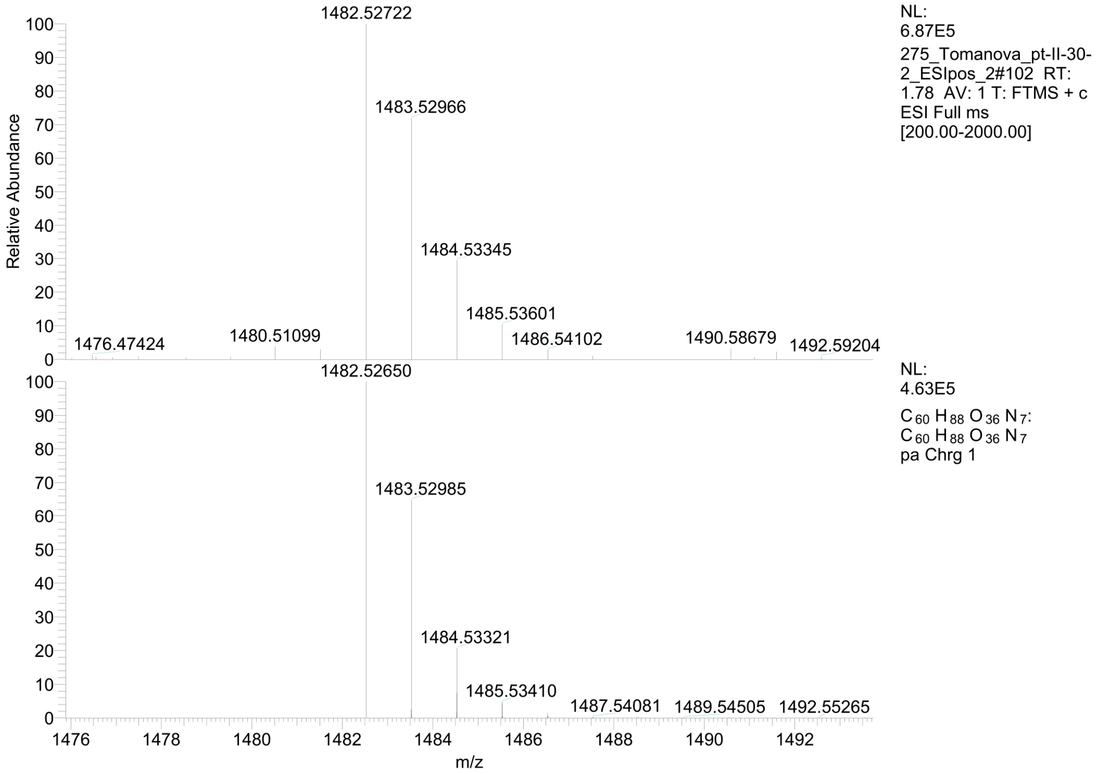

Figure S36. HR-MS spectrum of compound **3c**.

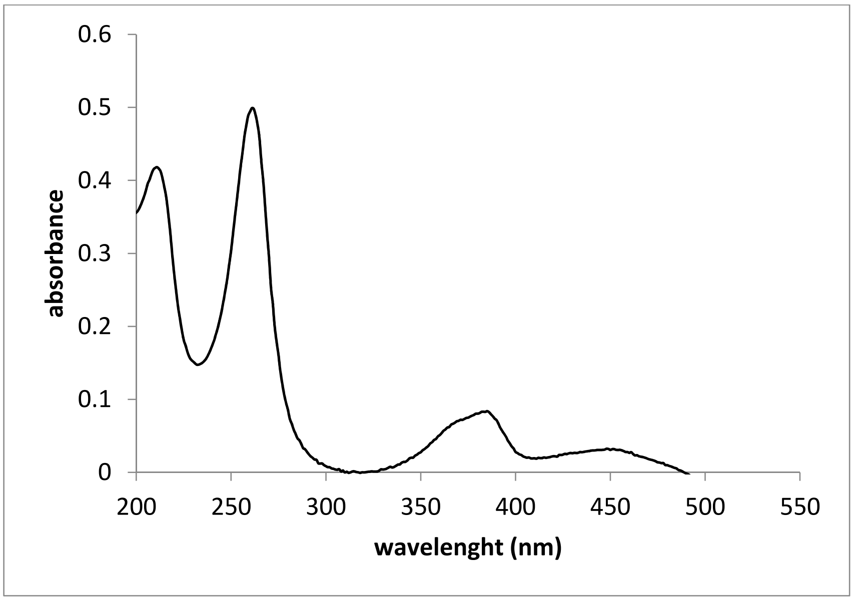

Figure S37. UV-VIS spectrum of compound **4**.

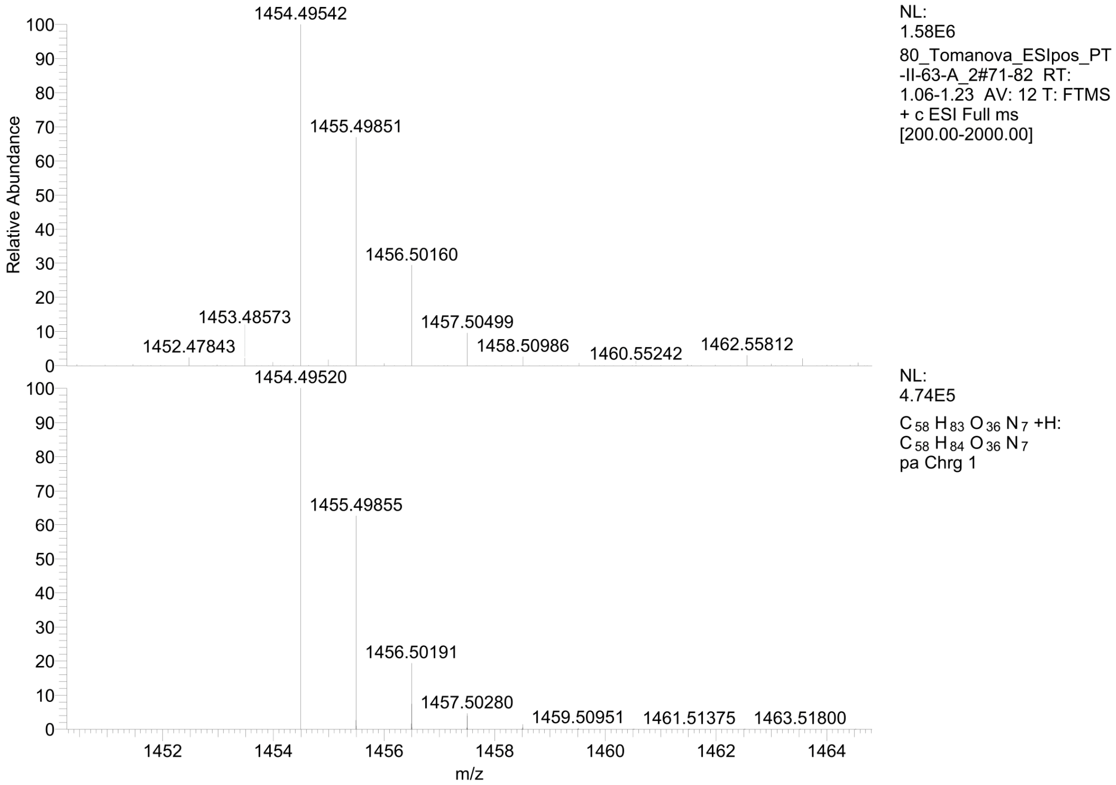

Figure S38. HR-MS spectrum of compound **4**.

5. Details on Stereoselective Oxidations

Table S5. H_2O_2-sulfoxidations catalyzed by conjugates **3** and **4** [a].

Sulfide	Conversion [b] [%]/ee [c] [%]			
	**3a**	**3b**	**3c**	**4**
n-$C_4H_9SCH_3$	quant./33	quant./25	quant./12	quant./16
n-$C_6H_{13}SCH_3$	42/39	28/26	23/23	53/12
n-$C_8H_{17}SCH_3$	9/11	8/9	5/14	8/8
n-$C_{10}H_{21}SCH_3$	34/10	2/-	1/-	2/14
t-$C_4H_9SCH_3$	quant./13	quant./11	quant./0	quant./0
c-$C_6H_{11}SCH_3$	77/26	49/12	50/5	24/20
$BnSCH_3$	59/35	31/32	40/30	38/0
p-TolylSCH_3	36/26	15/12	6/14	8/0
$PhSCH_3$	38/20	6/14	5/4	5/0

[a] Conditions: substrate (0.1 mmol), H_2O_2 (2.3 equiv.), phosphate buffer pH 7.5, 25 °C, catalyst loading 1 mol % (related to the substrate) if not stated otherwise; vigorous shaking for 1 h; [b] conversion determined by ^{1}H-NMR; [c] ee determined by HPLC on a chiral stationary phase (see below).

HPLC chromatograms obtained by measuring reaction mixtures after sulfoxidations with catalyst **3a** are shown for example. For details on sulfoxidations see main text.

Retention Time (min)	Relative Area (%)
23.62	75.45
34.87	24.55

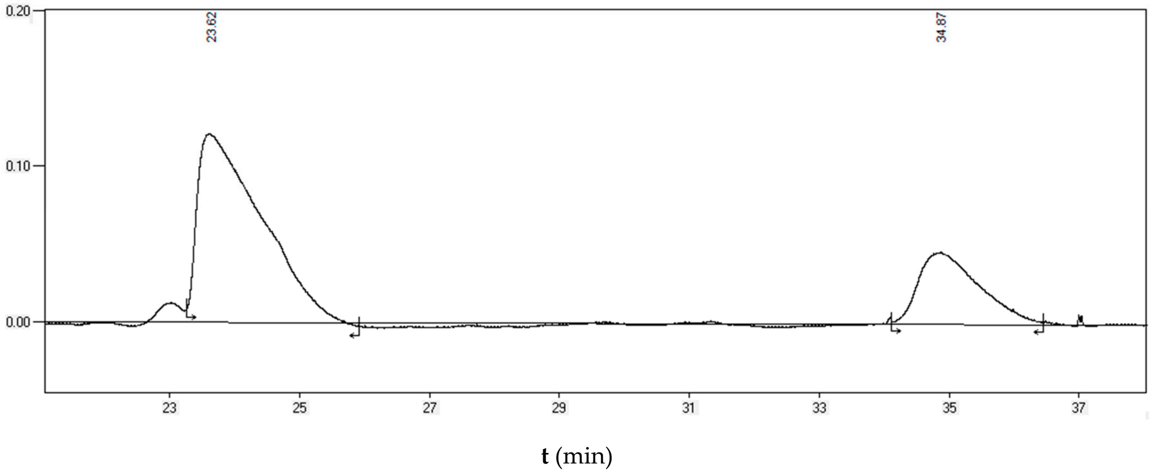

Figure S39. Butyl methyl sulfoxide (column AS-H, mobile phase heptane/propan-2-ol 80:20, flow 1 mL/min).

Retention Time (min)	Relative Area (%)
24.97	69.48
36.88	30.52

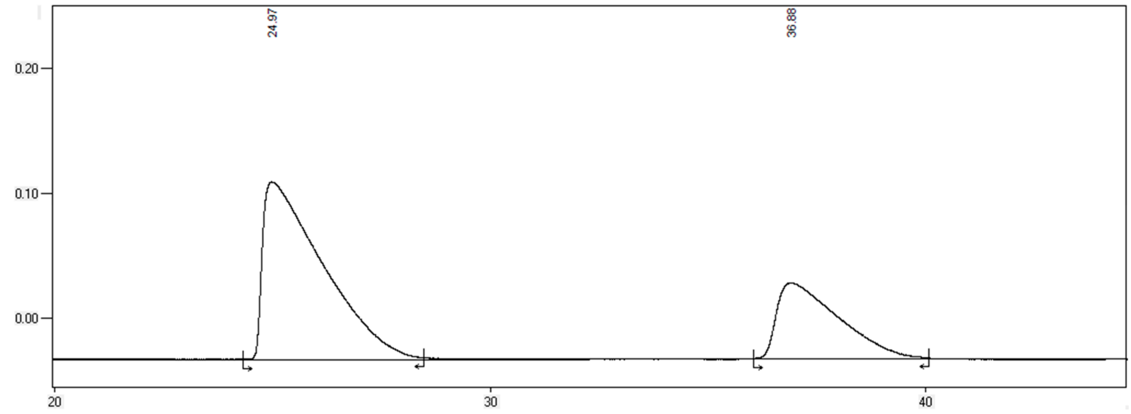

Figure S40. Hexyl methyl sulfoxide (column AS-H, mobile phase heptane/propan-2-ol 80:20, flow 1 mL/min).

Retention Time (min)	Relative Area (%)
16.96	55.30
23.00	44.70

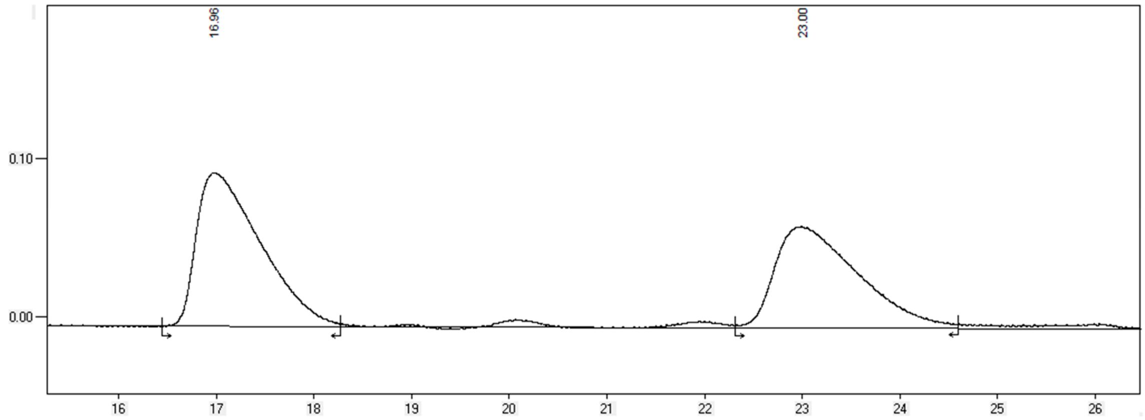

Figure S41. Octyl methyl sulfoxide (column AS-H, mobile phase heptane/propan-2-ol 80:20, flow 1 mL/min).

**Retention Time (min)**	**Relative Area (%)**
15.33	54.80
20.75	45.20

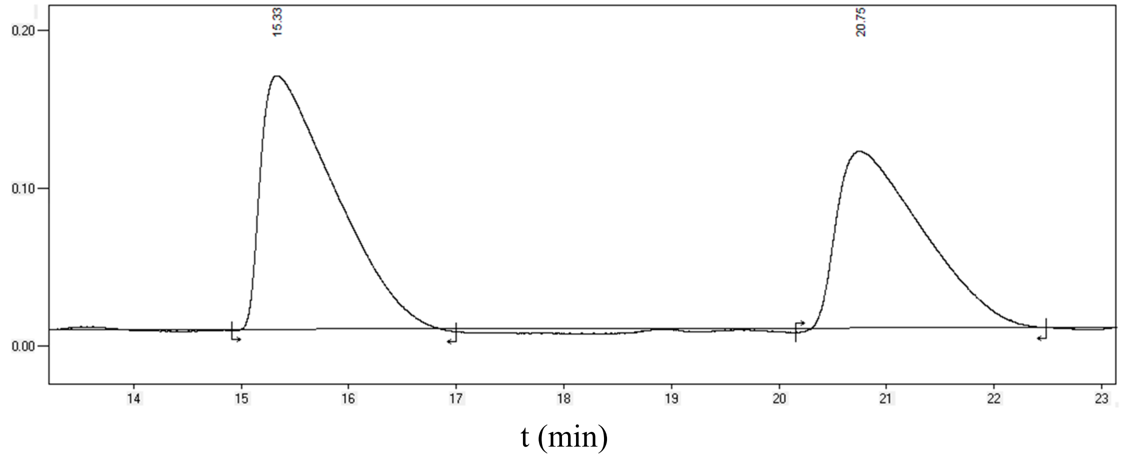

Figure S42. Decyl methyl sulfoxide (column AS-H, mobile phase heptane/propan-2-ol 80:20, flow 1 mL/min).

**Retention Time (min)**	**Relative Area (%)**
19.08	65.00
20.35	35.00

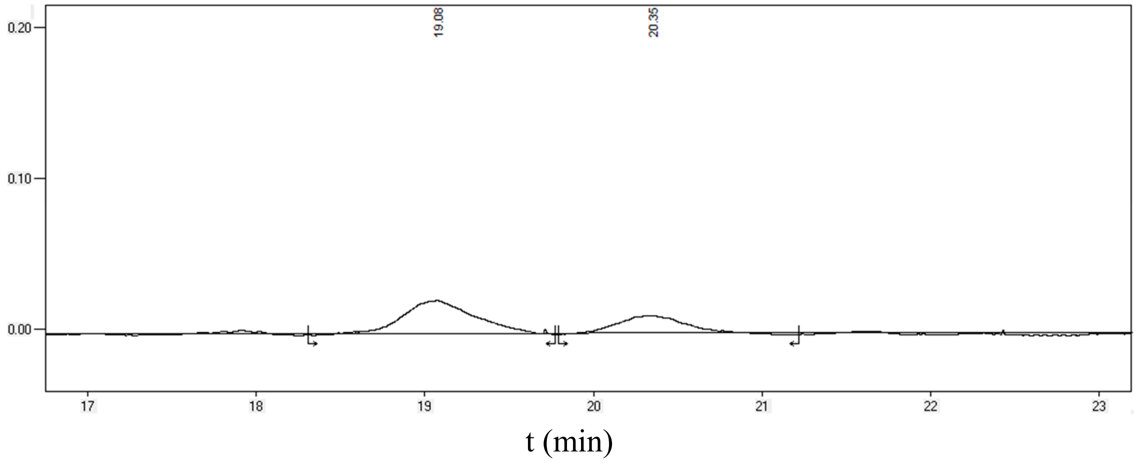

Figure S43. *t*-butyl methyl sulfoxide (column Celulose-4, mobile phase heptane/propan-2-ol 90:10, flow 1 mL/min).

Retention Time (min)	Relative Area (%)
21.19	62.75
23.54	37.25

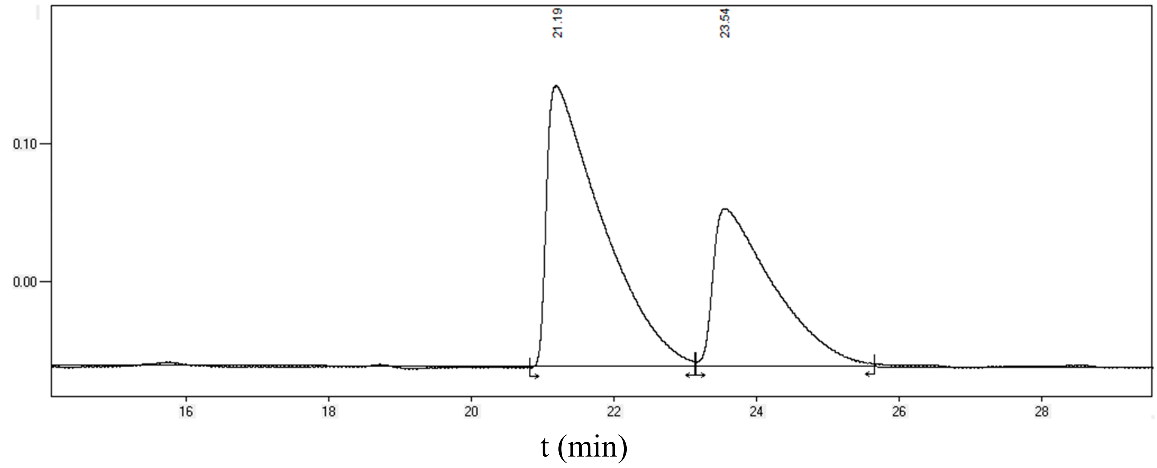

Figure S44. Cyclohexyl methyl sulfoxide (column Amylose-2, mobile phase heptane/propan-2-ol 90:10, flow 1 mL/min).

Retention Time (min)	Relative Area (%)
17.32	32.57
20.40	67.43

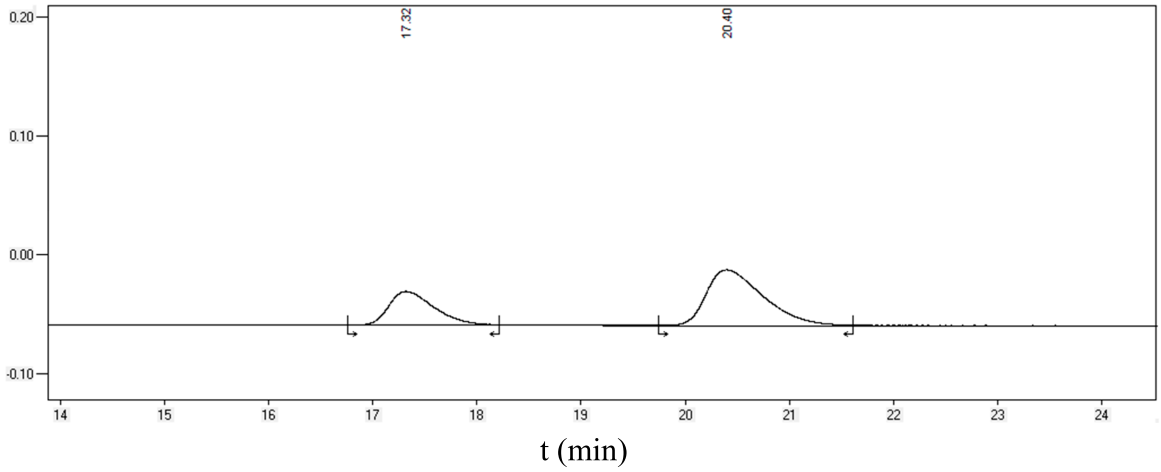

Figure S45. Benzyl methyl sulfoxide (column Celulose-4, mobile phase heptane/propan-2-ol 80:20, flow 1 mL/min).

Retention Time (min)	Relative Area (%)
14.72	36.81
16.51	63.19

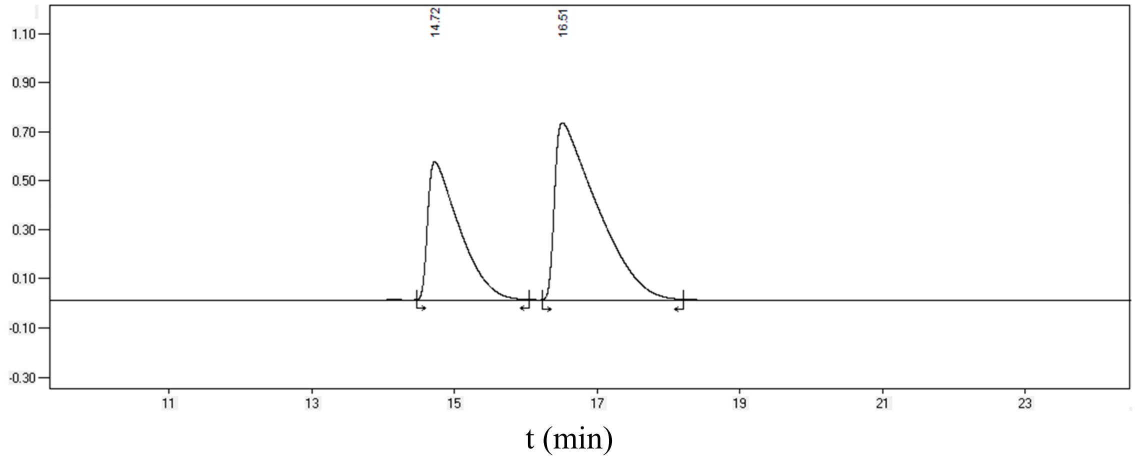

Figure S46. *p*-tolyl methyl sulfoxide (column Amylose-2, mobile phase heptane/propan-2-ol 80:20, flow 1 mL/min).

Retention Time (min)	Relative Area (%)
9.87	59.84
12.04	40.16

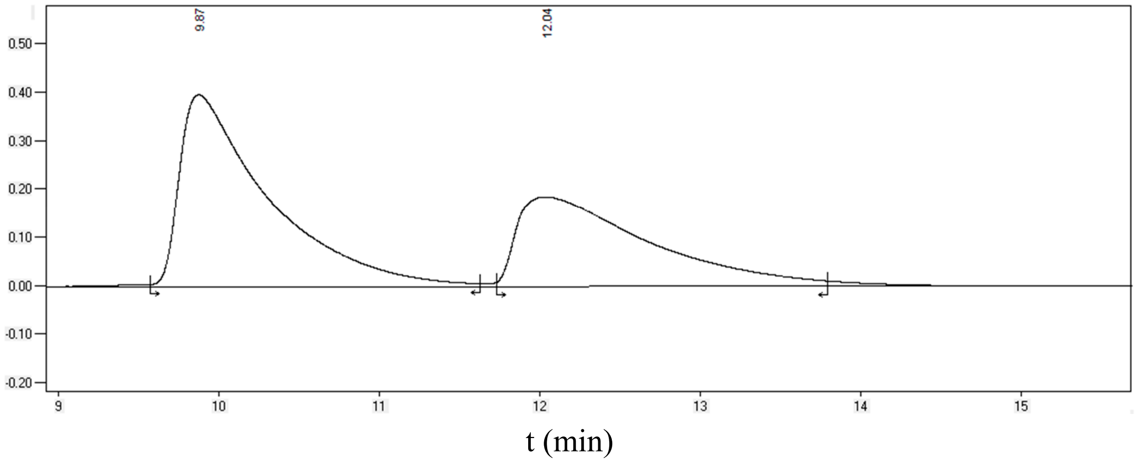

Figure S47. Fenyl methyl sulfoxide (column OD-H, mobile phase heptane/propan-2-ol 90:10, flow 1 mL/min).

6. Effect of Amide Bond on the Reactivity of Flavin-4a-Hydroperoxide

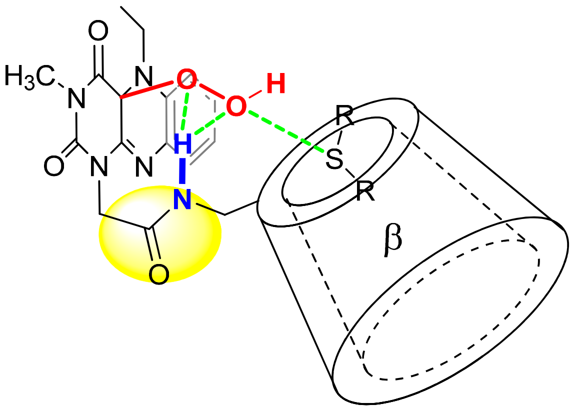

Figure S48. Illustration of hydrogen bonds influencing the reactivity of hydroperoxide function in oxygen transfer step from flavin hydroperoxide to a sulfide within sulfoxidations catalysed by **1**.
